# The Effect of a New Generation of Ankle Foot Orthoses on Sloped Walking in Children with Hemiplegia Using the Gait Real Time Analysis Interactive Lab (GRAIL)

**DOI:** 10.3390/bioengineering11030280

**Published:** 2024-03-16

**Authors:** Federica Camuncoli, Giorgia Malerba, Emilia Biffi, Eleonora Diella, Eugenio Di Stanislao, Guerrino Rosellini, Daniele Panzeri, Luigi Piccinini, Manuela Galli

**Affiliations:** 1 Department of Electronics Information Technology and Bioengineering, Politecnico di Milano, 20133 Milan, Italy; federica.camuncoli@mail.polimi.it; 2 Scientific Institute IRCCS Eugenio Medea, 23842 Bosisio Parini, Italy; 3 ITOP SpA Officine Ortopediche, 00036 Palestrina, Italy

**Keywords:** gait, GRAIL, hemiplegia, AFO, ankle foot orthosis, cerebral palsy

## Abstract

Cerebral palsy poses challenges in walking, necessitating ankle foot orthoses (AFOs) for stability. Gait analysis, particularly on slopes, is crucial for effective AFO assessment. The study aimed to compare the performance of commercially available AFOs with a new sports-specific AFO in children with hemiplegic cerebral palsy and to assess the effects of varying slopes on gait. Eighteen participants, aged 6–11, with hemiplegia, underwent gait analysis using GRAIL technology. Two AFO types were tested on slopes (uphill +10 deg, downhill −5 deg, level-ground). Kinematic, kinetic, and spatiotemporal parameters were analyzed. The new AFO contributed to significant changes in ankle dorsi-plantar-flexion, foot progression, and trunk and hip rotation during downhill walking. Additionally, the new AFO had varied effects on spatiotemporal gait parameters, with an increased stride length during downhill walking. Slope variations significantly influenced the kinematics and kinetics. This study provides valuable insights into AFO effectiveness and the impact of slopes on gait in hemiplegic cerebral palsy. The findings underscore the need for personalized interventions, considering environmental factors, and enhancing clinical and research approaches for improving mobility in cerebral palsy.

## 1. Introduction

Cerebral palsy (CP) is a non-progressive neurological disorder characterized by involuntary motor and posture impairments, arising from prenatal, perinatal, or postnatal central nervous system malformations or lesions [1]. The classification of CP is based on various factors, including limb involvement, resulting in subtypes such as hemiplegia, diplegia, and quadriplegia.

Hemiplegia manifests on the side of the body opposite to the cerebral damage. It presents pathological patterns like hypokinesia in half of the body, asymmetry, and altered muscle tone and strength, with increased muscle and joint stiffness. For instance, individuals with hemiplegia often exhibit asymmetries in their gait spatiotemporal parameters. This includes a reduced step length and stance phase duration, along with increased swing time on the affected side in comparison to the less affected side [2]. Gait patterns in children with hemiplegia vary based on the adopted walking strategies. Observations during walking revealed a reduced base of support, body weight shift towards the less affected side, retro-positioning of the affected side with semi-flexed hip and abducted lower limb, excessive lower limb flexion in the swing phase, and valgus-pronated or equino-varo supinated foot contact [3]. Increased muscular and joint stiffness in the stance phase results in hip flexion with knee recurvatum to facilitate heel contact with the ground [3]. Furthermore, under uphill and downhill walking conditions, individuals are likely to encounter greater difficulties [4], which can be mitigated with the aid of support devices.

To enhance walking, mobility, and therefore autonomy and independence, ankle foot orthoses (AFOs) are commonly prescribed [5,6]. AFOs are orthoses that act at the level of the patient’s lower limb, ankle, and foot, providing stability to the tibiotarsal joint and exerting biomechanical control above and below this joint [7], imposing a mechanical constraint.

Traditional AFOs have been associated with limitations such as restricted mobility, discomfort, and challenges in adapting to high temperatures, particularly during hot seasons. This is attributed to the extensive coverage of the leg with thermoplastic material, hindering skin breathability [8]. Additionally, there are several limitations and barriers to the everyday applicability of traditional AFOs, including their non-modularity, limited adaptability to varying daily activities, lack of comfort and wearability, and the necessity for device updates to accommodate growth and development. These factors contribute to the complexity of using AFO orthoses [9]. In contrast, the new generation AFO, specifically designed for sports activities, features a prefabricated carbon fiber posterior leaf spring that allows for improved breathability throughout the calf, reducing the percentage of the leg covered by thermoplastic material to only the popliteal area. This advancement aims to address the aforementioned limitations by offering enhanced flexibility, comfort, modularity, and biomechanical support [10,11], thereby potentially improving gait patterns in children with hemiplegic CP.

ITOP Officine Ortopediche designed a new-generation AFO by incorporating some structural modifications into an existing model known as the carbon modular orthosis (Ca.M.O.) [10]. This new design retained the modularity aspect and the ability to customize the orthosis according to the patient’s needs. Both models consist of three main components: a custom-made polypropylene shell for the calf and one for the foot, constructed from thermoplastic material, and a carbon posterior leaf spring with interface link elements connecting these two elements.

**Figure 1:**
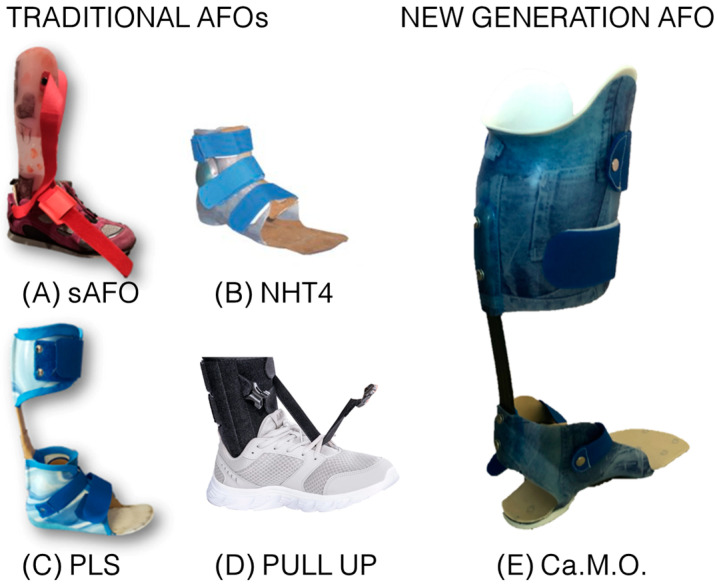
On the left are traditional AFOs. On the right is the new-generation AFO. (**A**) sAFO: solid ankle foot orthoses; (**B**) NHT4: Nancy Hylton T4; (**C**) PLS: posterior leaf spring; (**D**) Pull up; (**E**) Ca.M.O.: carbon modular orthosis.

Compared to the traditional Ca.M.O. orthosis, the one developed for this project (Figure 1E) features a different carbon spring stratification, with fewer layers of unidirectional fibers in favor of “X-pattern” fiber layers. This modification aims to enhance the orthosis’s flexibility, enabling it to adapt more effectively to various movements without imposing excessive restrictions. The rear heel area has an open design with a “reverse V” profile to accommodate the expansion of the adipose cushion located at the base of the heel during ground contact, effectively acting as a natural shock absorber. Additionally, a carbon plate has been inserted under the AFO’s sole to increase the stiffness of the AFO’s tip, which may improve responsiveness in physical activities. This design enables the accumulation of greater elastic energy during the first half of the stance phase when the foot contacts the ground, with an attempt to return a larger portion of it during the second half. The choice of the leaf spring stiffness from nine existing profiles was determined based on the anthropometric dimensions of the individuals using the “load index” calculation method [10].

Gait analysis is mandatory to assess AFOs’ effectiveness. However, limitations arise when using only optoelectronic systems and force platforms, including the inability to pre-determine walking speed, reduced number of analyzed gait cycles, and non-representative lab environments for daily living conditions [12,13].

To address these challenges, a comprehensive approach employing a treadmill, a moving platform, a stereophotogrammetric system, and a virtual reality environment mimicking sloped walking becomes essential. The Gait Real-time Analysis Interactive Lab (GRAIL) serves as a dedicated solution for gait analysis and training in challenging conditions, aiming to assess and improve gait patterns [4,14,15,16].

Previous research studies have explored various aspects of gait in children with CP. Choi and collaborators [16] studied barefoot walking in children with hemiplegic CP on different slopes, comparing the sagittal kinematics of the affected limb with the less-affected one. They concluded that affected and less affected limbs exhibited different adaptation patterns. Ma and coauthors [17] compared barefoot level and uphill walking in children with diplegia to a control group of typically developing children. Despite similar adjustments during uphill walking compared to the control group, uphill walking worsened gait abnormalities in children with CP. Van der Krogt et al., [18] conducted a study comparing self-paced treadmill walking to walking in a conventional gait lab in children with and without CP (participants opted for their personal low, flat-soled footwear, which included insoles or orthoses if regularly worn). They observed an increased stride width and shorter stride length on the treadmill compared to overground walking, with similar kinematic curves. However, children with CP exhibited increased gait deviation on the treadmill with respect to typically developing children.

Additionally, one study explored the feasibility of walking with AFOs using the GRAIL system in ten adult patients with flaccid plantarflexor and dorsiflexor muscle paresis in three slope conditions: uphill, downhill, and level-ground [19], considering only a few kinematics parameters such as ankle maximum dorsiflexion, ankle range of motion, and the knee maximum and minimum flexion during swing. Another study investigated the effect of three types of ankle foot orthoses (solid, articulated, and posterior leaf) on the gait pattern of 13 adult post-stroke patients during level-ground, uphill, and downhill surfaces using GAITRite focusing on spatiotemporal parameters [20].

To the best of our knowledge, our study is not the first one analyzing walking on the GRAIL of children with hemiplegia [16,18,21], but it represents a significant contribution, addressing a gap in the literature by analyzing walking in three conditions (i.e., level-ground, ascent, and descent) on the GRAIL with different AFO devices.

The need for this study arises from the necessity to understand whether the use of the new generation of AFO improves or does not improve walking on level, uphill, and downhill surfaces compared to the previous traditional devices.

The primary objective of this study was to compare hemiplegic CP children’s gait on the GRAIL under various conditions that may occur during walking in ordinary life (uphill, downhill, and level-ground) while wearing commercially available, typically used AFOs, or a new generation of AFO specifically developed for sports activities by ITOP Officine Ortopediche. The GRAIL was chosen for its ability to assess multistep gait even on incline/decline planes. The second objective was to compare, under the same AFO orthotic conditions, the differences in terms of gait kinematics and kinetics during level-ground, uphill, and downhill walking in children with hemiplegic CP, aiming to observe their management of walking on slopes while wearing AFOs. Comparing walking with orthoses on flat ground and uphill and downhill slopes is useful because it allows for assessing the orthoses’ performance in different situations, providing more comprehensive insights into their functionality and effectiveness in supporting the patient during various walking activities closer to an ecologic context.

## 2. Materials and Methods

### 2.1. Participants

The study, conducted as part of the GIFT—engineering for sport for all—project, obtained approval from the Ethics Committee of the Scientific Institute IRCCS Eugenio Medea (Bosisio Parini, Lecco, Italy) (protocol code 20/20—CE and date of approval 20 April 2020). All the participants’ parents/guardians read and signed the informed consent.

Eighteen children were enrolled based on the following inclusion criteria: (i) diagnosis of right or left hemiplegia due to CP, (ii) AFO users, (iii) aged 6–11 years old, (iv) Gross Motor Function Classification System (GMFCS) level I–II, (v) Modified Ashworth scale [22] score of less than or equal to 3 on the following muscle groups: triceps surae, hamstrings, and rectus femoris, and (vi) demonstrating hyperactivity of the triceps surae muscle (Table 1). The exclusion criteria from the study were as follows: (i) utilization of knee and hip orthoses (KAFO, HKAFO), (ii) dependence on assisted ambulation (usage of crutches, walkers, or other aids), and (iii) children who are non-compliant. The children were recruited by clinicians at the Scientific Institute IRCCS Eugenio Medea. After the enrolment, each child received a new custom-made AFO and a pair of orthopedic shoes of the correct size manufactured by ITOP Officine Ortopediche (Palestrina, Rome, Italy) and Duna (Falconara Marittima, Ancona, Italy), respectively.

### 2.2. Gait Data Collection

**Figure 2:**
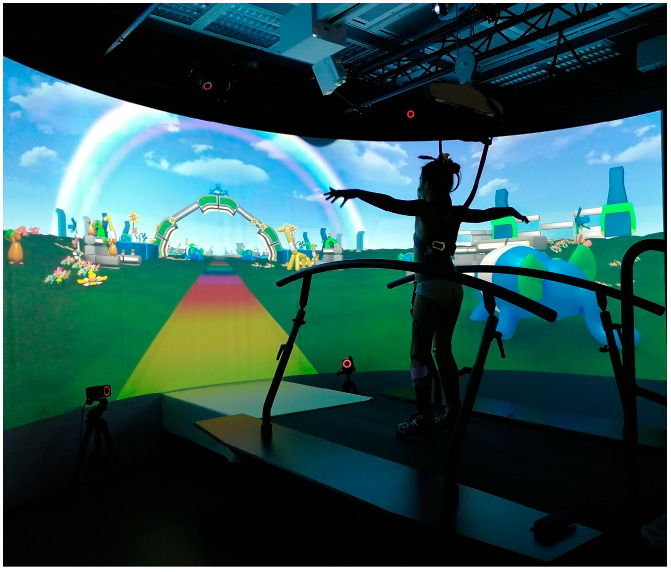
A patient on the GRAIL during the experiment.

Experiments were conducted using the Gait Real Time Interactive Lab (GRAIL) (Motek Medical B.V., Amsterdam, The Netherlands), a multi-sensor platform employing immersive virtual reality (Figure 2). The system incorporates ten optoelectronic motion analysis cameras (sample frequency 100 Hz) for kinematic data acquisition (Vicon, Oxford Metrics, Oxford, UK), a motion platform, and an instrumented dual belt treadmill (pitch of +/− 10°) with integrated force plates (sample frequency 1000 Hz) for kinetic data assessment. Safety measures included two lateral handrails and a harness.

Twenty-six reflective markers were placed on anatomical landmarks according to the human body lower limb model with trunk protocol (HBM-II) [23]: on the 7th cervical vertebra, on the 10th thoracic vertebrae, on the xiphoid process of the sternum, on the jugular notch of the sternum, on the left and right anterior superior iliac spines, on the left and right posterior superior iliac spines, on the lateral side of the left and right knee joint axis, half on the line between the left and right greater trochanter and the lateral side of the left and right knee joint axis, on the center of the left and right lateral malleolus, half on the line between the lateral side of the left and right knee joint axis and on the center of the left and right lateral malleolus, on the caput of the left and right 5th meta tarsal bone, on the joint line of the midfoot/toes, on the caput of the left and right 2nd meta tarsal bone, on the joint line of the midfoot/toes, and on the center of the left and right heel at the same height as the 2nd left and right meta tarsal marker.

Two conditions at three slope levels (level-ground 0°, uphill 10°, and downhill −5°) were tested: (i) walking with commonly used AFOs, (ii) walking with the newly prescribed AFO. Participants completed the conditions in a randomized order. A synchronized virtual reality environment showing a colorful pathway with balloon animals on the side was displayed on the 180° GRAIL screen to encourage the child’s performance (Figure 2).

After a 10 min adaptation period, the walking speed was maintained at a constant value approximating each participant’s preferred overground gait velocity. Twenty steps were recorded for each AFO condition at each slope (0°, 10°, and −5°). In total, each participant took 40 steps for each slope (20 steps for the affected side, 20 steps for the less affected side), wearing the typically used AFO. The entire protocol was then repeated with the new-generation AFO (20 steps for each limb and each condition). A comprehensive analysis of 120 steps for each AFO condition was performed.

### 2.3. Orthosis Description

The study compared the orthoses commonly used by patients to the new generation AFO developed by ITOP Officine Ortopediche based on a carbon modular orthosis (Ca.M.O). Participants used their traditional orthotic devices, previously prescribed by the physiatrist and utilized in their everyday life (Table 1). Thanks to this project, the patients tested a new-generation orthosis tailored to their anthropometrics and gait features for one month (Figure 1E). The types of orthoses used are described below:

- *Solid Ankle Foot Orthosis (sAFO)* (Figure 1A): sAFO consists of a single, rigid element, typically made of thermoplastic material, which completely blocks plantar-flexion and dorsiflexion of the ankle.
- *Nancy Hylton dynamic brace (NHT3/T4)* (Figure 1B): NH3/T4 is a type of orthosis designed to restore the foot to its correct anatomical position. There are various models of this brace with different containment heights; and specifically the T3 orthosis, used when containment of the tibiotarsal joint is required, includes the malleoli but remains open on the instep, while the T4 orthosis also wraps around the instep to block the entire malleolar joint complex.
- *Posterior Leaf Spring (PLS)* (Figure 1C): PLS is a different type of AFO in which the trimline of the calf creates a posterior spring-like element that allows a slight plantar-flexion and dorsiflexion movement of the ankle, it prevents the development of ankle contractures; it allows initial contact with the heel, and in some cases also a slight plantar-flexion which partially restores the first rocker; it also normalizes the dorsiflexion of the ankle in the stance phase, thus also improving the movement of the ankle in the stance phase.
- *Pull Up* (Figure 1D): The pull up is a dynamic equine foot support made of fabric with Velcro closures, with elastic tie-rod quick release.
- *Ca.M.O.* (Figure 1E): Ca.M.O. consists of three main components: a custom-made polypropylene shell for the calf and one for the foot, constructed from thermoplastic material, and a prefabricated carbon posterior leaf spring with interface link elements connecting these two elements [10]. This design aims to achieve greater deformation under the same applied load, facilitating adaptation to activities like running.

### 2.4. Data Processing

For the kinematic calculations, the joint centers of the hip, knee, and ankle have been defined, as reported in the Motek manufacturer’s manual. A predictive method was utilized to determine the hip joint center (HJC) following the Harrington equations [24]. These equations consist of linear regression models designed to predict the HJC’s location based on the pelvic width and pelvic depth. Pelvic width is measured as the distance between the two ASIS markers, while pelvic depth is defined as the distance between the midpoints of the line segments connecting the two ASIS and the two PSIS. The knee joint center is assumed to be the midpoint between the lateral and medial epicondyles, while the ankle joint center is assumed to be the midpoint between the lateral and medial malleoli. All these calculations were embedded in the Motek pipeline.

Kinematic, kinetic, and spatiotemporal data were pre-processed using the gait online analysis tool (GOAT). The real-time filtering of GRAIL data was performed employing a low-pass 2nd-order Butterworth filter with a cutoff frequency of 6 Hz for the kinematic data and a low-pass 2nd-order Butterworth filter with a cutoff frequency of 6 Hz for kinetic data. Subsequently, all the data were time-normalized into 100 samples representing the gait cycle (GC). GOAT software 4.2 allowed for the manual deletion of individual missteps, such as gait cycles that had a foot placement on only one of the force platforms or when passive markers were not visible. The total number of strides correctly recorded, with an average of 18 gait cycles for each child side, were exported in a .csv file. Then, data were exported and analyzed through a custom Matlab^®^ script (MATLAB R2021b, The MathWorks, Inc., Natick, MA, USA). The two limbs, namely the affected and the less affected sides, were analyzed separately, and the mean and standard deviation of the time series of the selected strides were computed for each child. Kinematics for the trunk, pelvis, and hip were computed in the sagittal, frontal, and transversal planes, while knee and ankle angles were analyzed in the sagittal plane. Kinetic parameters included hip, knee, and ankle moments and powers. Spatiotemporal parameters such as stance phase duration expressed as a percentage of the gait cycle (%GC), stride duration (s), stride and step length (m), and step width (m) were also calculated. All these variables were calculated using a pipeline within the GOAT software provided by Motek.

### 2.5. Statistical Analysis

Statistical parametric mapping (SPM) [25] was used to test the differences in the downhill, level, and uphill gait kinematics and kinetics between the commonly used and new AFOs. The same statistical method allowed for the comparison of the three slopes under the same orthotic condition.

Data normality was confirmed using the D’Agostino–Pearson K2 test. A two-tailed paired t-test was used to compare the new-generation AFO and the commonly used one across the three conditions (downhill, level-ground, and uphill). Additionally, a repeated-measure ANOVA 2 (side: affected, less affected) × 3 (conditions: uphill, downhill, level-ground) design was applied, followed by a post-hoc test to evaluate the three slope levels while keeping the orthotic conditions consistent. The level of significance was set at 0.05 with Bonferroni correction. For spatiotemporal parameters, due to the non-normality of the dataset, a Wilcoxon test was performed with a level of significance set at 0.05 to detect the differences in the same condition between the commonly used AFO and the new one. The comparison among the three slopes was conducted using a Friedman test (α = 0.05) followed by post-hoc tests.

## 3. Results

### 3.1. Participants

Eighteen children were initially recruited for the study (11 males and 7 females, age: 8.0 ± 1.5 years, height: 129.3 ± 7.3 cm, body mass 27.4 ± 5.3 kg (mean ± SD). One participant was excluded from the analysis due to the inability to perform the tests and another one was excluded only from the downhill walking analysis. Therefore, seventeen children were included for both conditions for uphill and level-ground walking, and sixteen individuals for downhill walking. Participants’ characteristics, including gender, age, body mass, height, diagnosis, type of AFO, and Gross Motor Function Classification System level, are reported in Table 1.

### 3.2. New Generation AFO vs. Commonly Used AFO

**Figure 3:**
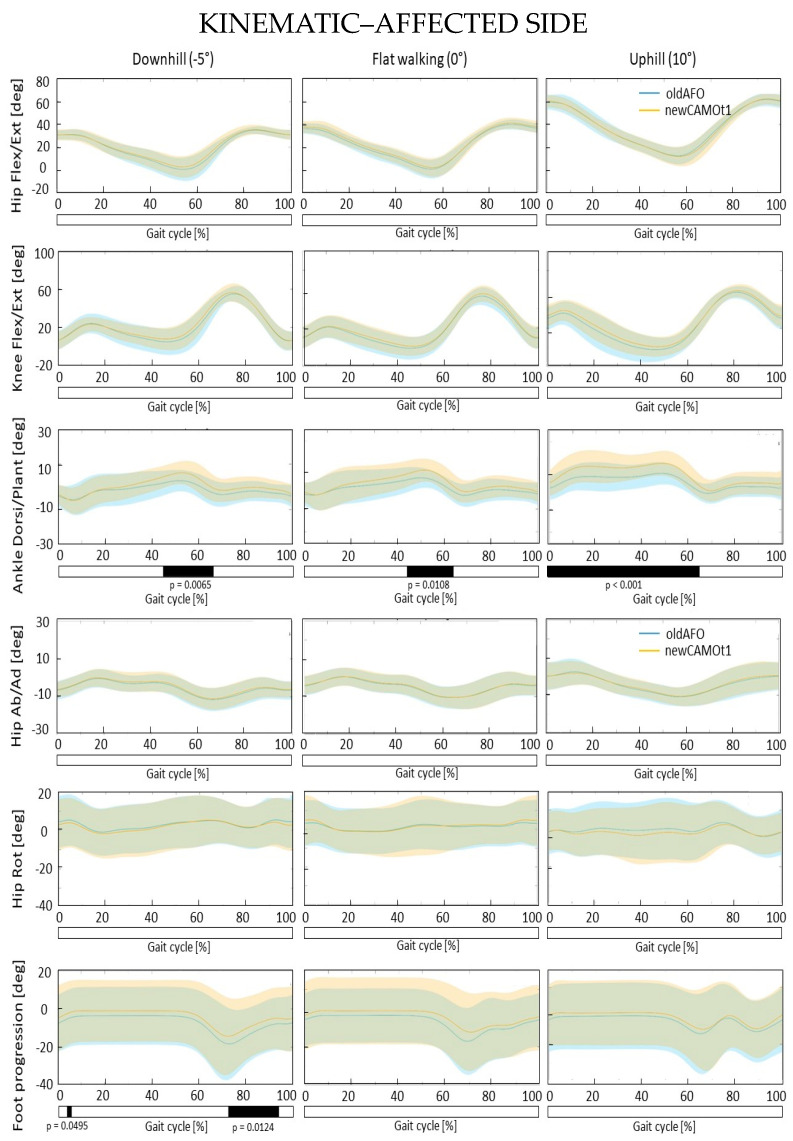
Mean and standard deviation of the kinematics of the affected hip, knee, and ankle joints in the sagittal plane, hip abduction and adduction, rotation, and foot progression for commonly used AFOs (depicted in blue, oldAFO) and the new AFO (depicted in orange, newCAMOt1) after the adaptation period with the related SPM analysis. *X*-axis (0–100% gait cycle), *Y*-axis (degrees). Hip flexion extension (+)/(−), knee flexion extension (+)/(−), ankle dorsi-plantar-flexion (+)/(−). Hip abduction adduction (−)/(+), hip internal external rotation (+)/(−), foot progression: internal external (+)/(−).

SPM{t} statistically significant changes between the two types of orthoses (i.e., typically used vs. new generation) were observed in the ankle dorsi-plantar-flexion of the affected leg in all three conditions (downhill: 43–65% GC, *p* = 0.007; level-ground: 43–62% GC, *p* < 0.011; uphill: 0–65% GC, *p* < 0.001). For the affected side during downhill walking, differences were found also in the foot progression angle immediately after heel contact and in the swing phase (5–7% GC, *p* = 0.049; 72–95% GC, *p* = 0.012), as well as in trunk rotation (55–57% GC, *p* = 0.049). On the contralateral side, hip rotation differed only during downhill walking (65–66% GC, *p* = 0.049). No statistically significant differences were found in terms of kinematics and kinetics for all other computed gait curves (Figure 3) (Supplementary Materials). Regarding spatiotemporal parameters, from the comparison between the traditional and the new AFOs, differences were found only in the affected limb, specifically in stride length (*p* < 0.001) for the downhill gait, and stance phase (*p* < 0.001) and stride time (*p* < 0.001) for the uphill gait. The parameters showing differences exhibited greater values with the new-generation AFO compared to the traditional one (Table 2).

### 3.3. Uphill vs. Downhill vs. Ground-Level Walking

**Figure 4:**
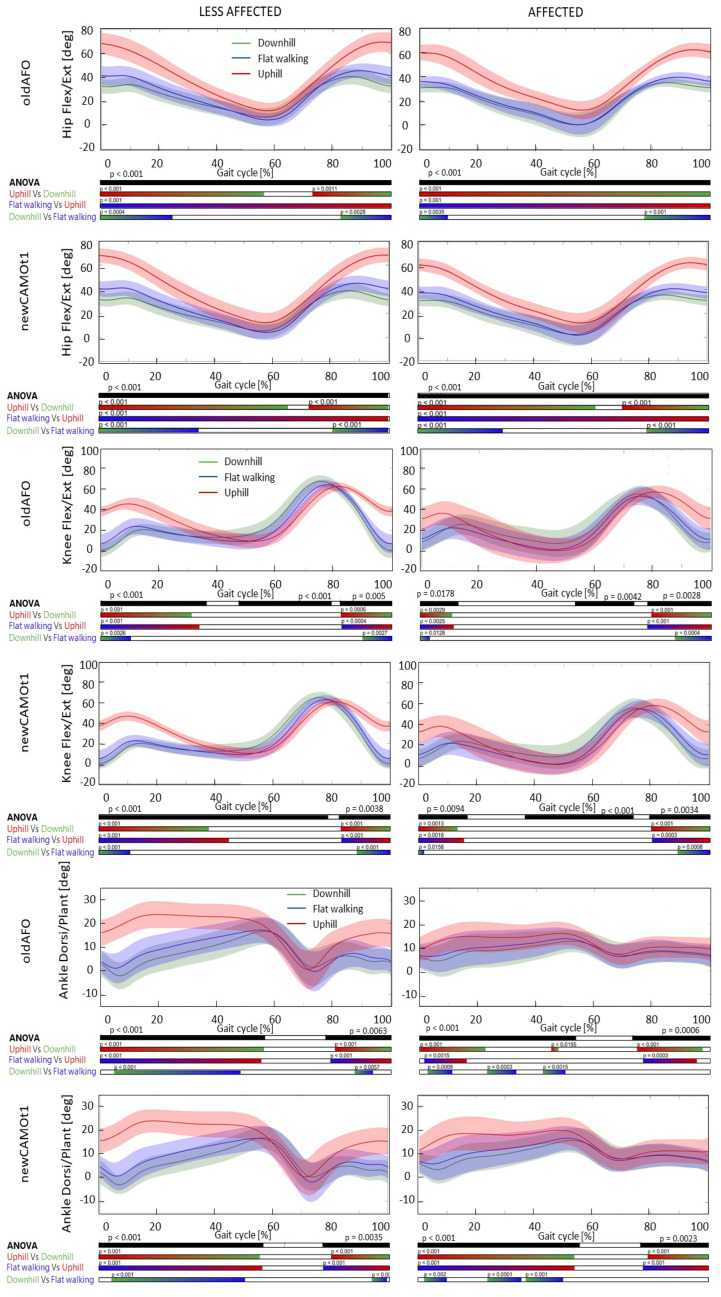
Mean and standard deviation of the kinematics of the hip, knee, and ankle joints in the sagittal plane for the affected and less affected sides with commonly used AFOs (oldAFO) and the new AFO (newCAMOt1) during downhill (in green), level-ground walking (in blue), and uphill (in red) conditions, along with the corresponding SPM analysis. *X*-axis (0–100% gait cycle), *Y*-axis (degrees). Hip flexion extension (+)/(−), knee flexion extension (+)/(−), ankle dorsi-plantar-flexion (+)/(−).

**Figure 5:**
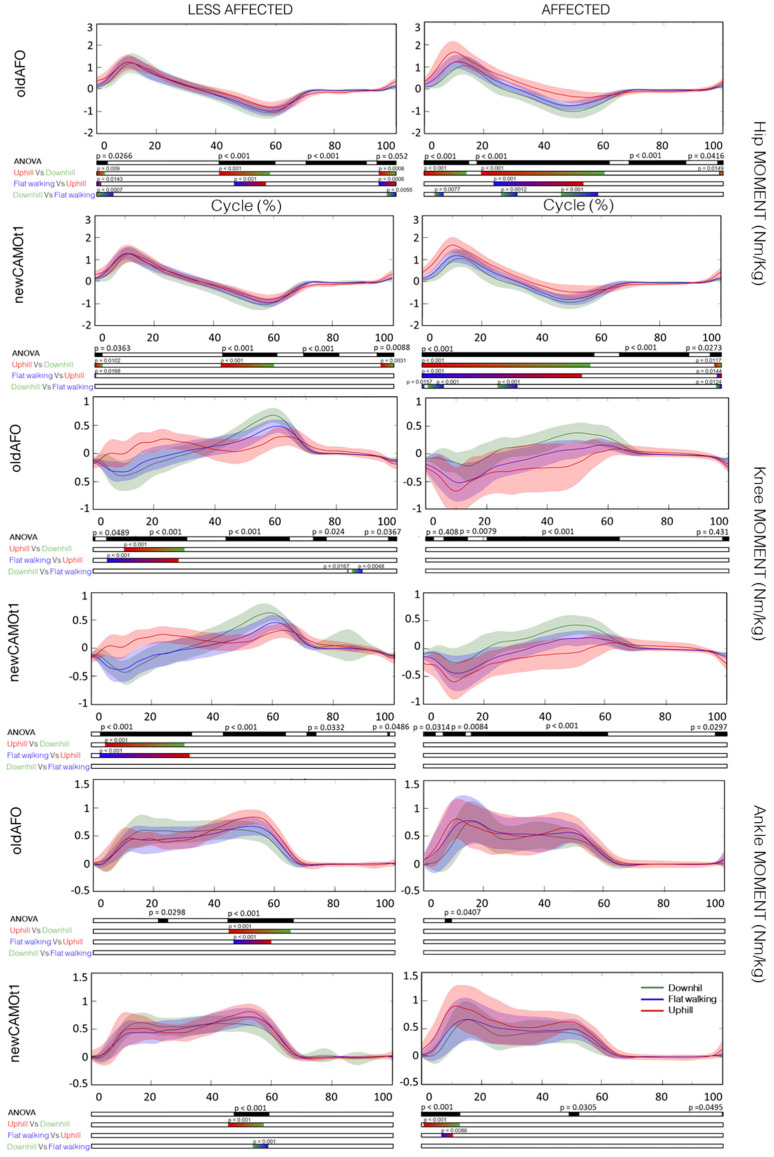
Mean and standard deviation of the moment of the hip, knee, and ankle joints in the sagittal plane for the affected and less affected sides with commonly used AFOs (oldAFO) and the new AFO (newCAMOt1) during downhill (in green), level-ground walking (in blue), and uphill (in red) conditions, along with the corresponding SPM analysis. X-axis (0–100% gait cycle), Y-axis (Nm/kg). Hip flexion extension moment (−)/(+), knee flexion extension moment (−)/(+), ankle dorsi-plantar-flexion moment (−)/(+).

**Figure 6:**
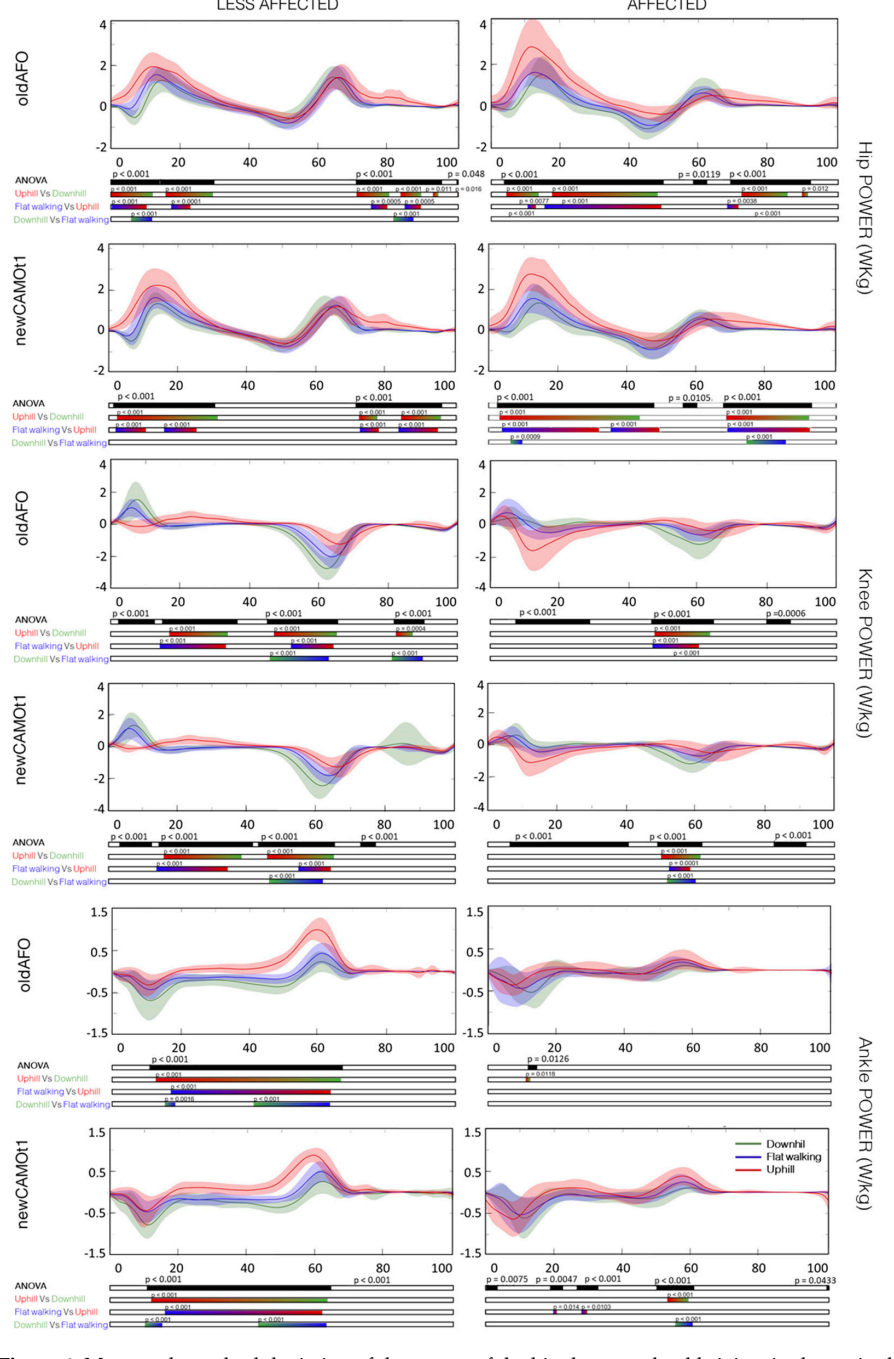
Mean and standard deviation of the power of the hip, knee, and ankle joints in the sagittal plane for the affected and less affected sides with commonly used AFOs (oldAFO) and the new AFO (newCAMOt1) during downhill (in green), level-ground walking (in blue), and uphill (in red) conditions, along with the corresponding SPM analysis. X-axis (0–100% gait cycle), Y-axis (W/kg). The power is positive when the body generates energy through concentric muscle activity. The power is negative when the body absorbs energy through eccentric muscle activity or elongation of soft tissue.

Comparing the three conditions, upslope, downslope, and level-ground, with the same type of orthosis, statistically significant differences were found in all analyzed time series, particularly evident in the post-hoc tests results depicted in Figure 4, Figure 5 and Figure 6. Neither the commonly used nor the new-generation AFOs altered the differences among the three slopes.

Regarding spatiotemporal parameters, a statistically significant difference in step length was found for the affected side with both types of orthoses between downhill and uphill walking (*p* < 0.001). In uphill walking, both traditional and new-generation AFOs showed an increased step length compared to downhill walking for the affected side. With the new AFO, the duration of the stance phase significantly differed between downhill and uphill walking (*p* < 0.001), being shorter during downhill walking. For the less affected limb, differences were observed with the new orthosis in the duration of the stance phase between level-ground and downhill walking (*p* < 0.001), as well as between downhill and uphill walking (*p* < 0.001), exhibiting an increasing trend from downhill, ground-level, and uphill walking. With traditional AFOs, differences in the stance phase were observed between downhill and uphill walking and level-ground and uphill walking, being shorter during downhill walking, greater during level-ground walking, and maximum during uphill walking (Table 3).

## 4. Discussion

The study was motivated by the need to assess whether the new generation of AFOs may enhance or not enhance walking performance in hemiplegic CP children across flat, uphill, and downhill terrains compared to traditional devices, addressing a gap in the literature regarding gait analysis with orthopedic AFOs under diverse conditions.

To address this gap, the study pursued a dual objective. Firstly, it aimed to compare commonly used AFOs with the new-generation AFO during downhill, level-ground, and uphill walking. This comparison is intended to validate the performance of the new AFO across various gait conditions, particularly focusing on significant differences in the sagittal kinematics and kinetics of the hip, knee, and ankle joints. Notably, the new AFO enabled in the affected side greater dorsiflexion angles during terminal stance and initial swing phases in downhill and level-ground walking, while in the uphill gait, greater dorsiflexion angles were maintained throughout the stance and initial swing phases. Although the orthosis allows for greater dorsiflexion, it may not appear to alter the gait kinetics in terms of moments and powers at the hip, knee, and ankle levels. Importantly, no compensatory mechanisms in either trunk and lower limbs kinematics and kinetics were detected (Supplementary Materials). However, it is important to note that these observations are preliminary, and further investigation is needed to confirm their significance.

Additionally, only during downhill walking were the following differences observed in terms of kinematics between the new device and traditional ones. The foot progression angle immediately after heel contact and in the swing phase was altered. The new device may allow for reduced external rotation of the lower limb (including the pelvic, hip, knee, ankle joints, and foot segment) during the mid-swing phase. Greater hip external rotation and trunk external rotation were observed with the new AFO. However, these differences were found to be statistically significant only for small portions of the gait cycle, suggesting that they may not have biomechanically significant implications for the walking motor pattern. In the comparison between traditional and new-generation AFOs, more significant changes in kinematics (ankle plantar-flexion angle, trunk rotation, foot progression) were observed in downhill walking compared to uphill walking, despite the downhill slope being halved (−5 degrees) compared to the uphill slope (+10 degrees).

Regarding spatiotemporal parameters, changes were observed for the affected limb. Specifically, during uphill walking, an increase in the stance phase duration was observed with the new AFO, allowing a prolonged weight-bearing period on the affected limb without the need to rely excessively on the less affected limb. An increase in stride duration was recorded when comparing the typically used AFO with the new one. Consequently, using the new orthosis increased both the length and duration of the stride, which also increased the duration of the stance phase percentage. In this latter metric, it was observed that there was a gain of one percentage point as it approached the percentage duration of the stance phase of the less affected limb. No statistically significant differences were found for level-ground walking. However, during downhill walking, there was an increase in step length with the new AFO. Typically, during downslope walking, there was a tendency to shorten the gait cycle to enhance stability [16,26,27,28,29,30,31]. However, in our case, the observed increase in stride length suggests that the new orthosis may indeed provide greater stability.

On the second front, the study explored walking on the treadmill at different slopes wearing the same AFO. The most significant kinematic changes were observed during uphill walking. This comparison was conducted using the same orthosis while assessing various inclines. It is suggested that the significant differences observed could be attributed to the chosen steeper incline angle (+10°), which is higher than the decline angle (−5°) and is asymmetric with respect to level-ground walking. The decision to limit the decline to only 5 degrees was made to maintain a protocol feasible for all children while ensuring safe conditions.

Uphill walking with both types of AFOs (i.e., the newly developed and the commonly used one) was associated with an increased flexion at the hip, knee, and ankle joints at initial contact, throughout the stance phase, and in the late swing phase for both the affected and less affected sides when compared to level-ground and downslope walking. Regarding hip internal and external rotation, no statistically significant differences were found with either AFO on either side. On the other hand, a significant difference in hip abduction–adduction was observed between uphill, level-ground, and downhill walking for the affected side at the beginning of the stance phase and in the terminal phase of the swing. Specifically, uphill walking resulted in greater hip adduction compared to level walking, whereas downhill walking resulted in reduced hip adduction. Additionally, for the progression of the affected foot, a difference was observed between uphill and level-ground or downhill walking during the mid-swing phase. Uphill walking exhibited a decrease in internal foot progression, whereas downhill walking showed an increase compared to level-ground walking.

Pelvic tilt and trunk inclination in the sagittal plane increased compared to ground-level walking and downslope walking. With the new AFO, the pelvic tilt during downhill walking differed throughout the entire gait cycle compared to level-ground walking; this difference was not observed with the previous commonly used AFO (Supplementary Materials—Figure S12).

The peak trunk obliquity and pelvic obliquity were greater during uphill walking compared to level-ground and downhill walking (Supplementary Materials—Figure S12). Minor differences were found in trunk and pelvic rotation among the three slopes as stated by Lay and co-authors [32].

Our findings, observed while wearing the AFO, align with prior studies analyzing both barefoot and shod walking on various populations, including healthy adults [33,34,35] [32], children with spastic diplegia, and typically developed ones [16,17,36,37,38,39]. Specifically, a detailed kinematic analysis without employing an SPM approach revealed how children with CP increased pelvic tilt, pelvic and trunk obliquity, knee flexion at initial contact, ankle dorsiflexion peak at initial contact, and ankle maximum dorsiflexion during uphill walking [17].

However, Choi and co-authors employing an SPM analysis revealed that the major changes in hip, knee, and ankle kinematics during uphill walking without AFOs in the barefoot condition occurred at initial contact and in the late swing phase for all three joints, while during the stance phase, changes were observed only for the hip and ankle [16]. Specifically, a greater tendency for flexion was observed, corroborating the outcomes obtained in our study (Figure 4, Figure 5 and Figure 6). Additionally, in downhill walking, differences in hip, knee, and ankle flexion extension were found only during the initial swing phase [16]. Except for this last observation regarding downhill walking, all other results are confirmed for both the affected and less affected sides when wearing the AFOs, both the commonly used ones and the new AFO developed for this project (Supplementary Materials).

Notably, several gait parameters were significantly influenced by the walking slope angle, with uphill walking probably having a more pronounced impact on gait kinematics compared to downhill walking as demonstrated by other studies. Interestingly, it was shown that gait coordination parameters remained unaffected, indicating the robust nature of gait asymmetry, left-right coordination, and gait variability [4]. The gait coordination parameters were as follows: the gait asymmetry assessed by comparing swing times between the legs, specifically computed as the absolute value of the natural logarithm of the ratio between left and right swing durations, multiplied by 100; the gait variability represented by the stride time coefficient of variation; and the phase coordination index which measures how well both sides of the body coordinate during walking, by checking how consistently and accurately they alternate steps between left and right [40].

In the affected limb, both the commonly used and newly developed AFOs exhibited an increase in peak hip moment during uphill walking compared to level-ground and downhill walking. Conversely, in the less affected limb, hip moment showed a comparable trend across all three conditions. At the knee joint, no differences were observed between level-ground, downhill, and uphill walking in the affected limb with either type of orthosis. However, in the less affected limb, the knee moment in a mid stance was a flexion moment during level-ground and downhill walking but an extensor moment during uphill walking.

Regarding ankle dorsi-plantar-flexion moment in the affected limb, no significant differences were found between level-ground, downhill, and uphill walking when wearing commonly used AFOs. However, with the new AFO, an increase in the first peak ankle plantar-flexion moment during uphill walking compared to level-ground and downhill walking was observed. For the contralateral less affected limb, the ankle plantar-flexion peak moment during push-off differed statistically among the three analyzed conditions.

Analyzing power, no significant differences were found at the ankle level in the affected limb with both orthoses. However, an increase in ankle power generation during the initial stance and the push-off was observed in the less affected limb when comparing the three slopes. Knee joint absorbed power may have played a more significant role during downhill walking compared to uphill walking, particularly during push-off. Additionally, the hip joint may have continued to be a driving force. In uphill walking, hip joint power generation differed statistically from that in level-ground and downhill walking during the stance phase for both the affected and less affected limbs when wearing both orthoses. This probably suggests possible compensatory mechanisms at the hip due to limited involvement of the tibiotalar joint [21].

It is noteworthy that only the study by Ma et al. analyzed both level-ground and uphill walking kinetics in children with CP [17]. Unlike our analysis, they found a decrease in the hip extension moment peak during the loading response phase. This suggests that the observed increase in the hip extension moment between 5% and 12% of the gait cycle in our study could be attributed to the presence of the AFOs. Consistent with the study of Ma et al., no variations were found in the knee moment [17]. However, previous studies reported an increase in the peak hip and knee extensor moments and ankle plantarflexor moments during uphill walking compared to level-ground walking [32].

Conversely, Ma et al., [17], observed a reduced ankle plantar-flexion moment during uphill walking compared to level-ground walking, while the presence of the AFOs seems to confirm a similarity between uphill and level-ground ankle plantar-flexion moments in the affected limb. Meanwhile, an increase in the ankle plantar-flexion moment was noted for the less affected limb with both the commonly used and newly developed AFOs. This disparity could be attributed not only to the presence of the AFOs but also to the recruited population. Ma and collaborators analyzed a group of children diagnosed with bilateral or diplegic CP, while our target population had hemiplegic CP [17].

However, it is imperative to acknowledge the study’s limitations. Firstly, the sample size was limited, warranting the inclusion of a more extensive patient cohort for robust analysis. The sample size of 18 patients was determined based on feasibility constraints and the available resources. Given the specialized nature of our study population and the resources available for recruitment and data collection, recruiting a larger sample size was not feasible within the scope of the study. Secondly, it is important to underline that our results are preliminary and may lack generalizability. Additionally, the absence of a control group for comparing the obtained 3D biomechanical analysis is a notable limitation. It is crucial for future research endeavors to address these limitations by expanding participant numbers and incorporating appropriate control groups for comprehensive validation. Moreover, investigating the long-term effects of customized AFOs on the overall mobility and quality of life in children with hemiplegia is essential for gaining deeper insights into the efficacy of orthotic interventions. It is crucial to be mindful of the potential type I errors associated with the numerous statistical tests performed on potentially correlated variables.

This is the first comprehensive 3D kinematic and kinetic analysis examining gait in children with hemiplegic CP, comparing commercially available AFOs with a new AFO developed for sports applications within a virtual reality environment. However, further clinical studies are needed to deeper understand the role of AFOs in sloped walking.

## 5. Conclusions

In this study, we investigated the effects of AFOs typically used by patients and new-generation AFO on the gait patterns of children with hemiplegic CP in various walking conditions: uphill, downhill, and level-ground. Utilizing the GRAIL system, which incorporates immersive virtual reality, a motion platform, and optoelectronic motion analysis, we compared the performance of commercially available, previously prescribed AFOs with a new generation of AFOs developed specifically for sports activities.

Our findings revealed significant differences between the two types of AFOs, particularly in ankle dorsi-plantar-flexion, foot progression angle, trunk rotation, and hip rotation during downhill walking. The spatiotemporal parameters also varied, with differences noted in stride length for the downhill gait and stance phase and stride time for the uphill gait. These results suggest that the new AFO has a positive impact on specific aspects of gait in children with hemiplegia, especially during challenging walking conditions.

Furthermore, our study compared the participants’ gait across different slopes (uphill, downhill, and level-ground) under consistent orthotic conditions. The analysis revealed significant differences in various kinematic and kinetic parameters, emphasizing the impact of slope variations on gait patterns in children with hemiplegic CP. However, neither the typically used nor the new-generation AFOs altered the differences among the three slopes. These findings underscore the importance of considering environmental factors, such as terrain incline, when assessing gait in individuals with motor impairments.

In summary, our research contributes valuable insights into the effectiveness of different AFO designs and the influence of slope variations on the gait of children with hemiplegic CP. By employing advanced technology like the GRAIL, we have enhanced our understanding of the complex interactions between orthotic interventions, environmental factors, and gait adaptations in pediatric patients. These findings have implications for clinicians and researchers working to improve mobility and quality of life for individuals with CP, highlighting the need for personalized and context-specific interventions tailored to the unique challenges posed by different walking conditions.

## Figures and Tables

**Table 1:** Participants’ characteristics of gender, age, body mass, height, affected side due to hemiplegic CP, and typically used AFO and GMFCS are reported. NHT3/4 = Nancy Hylton T3 and T4; sAFO = solid ankle foot orthosis; PLS = posterior leaf spring; GMFCS: Gross Motor Function Classification System.

ID	Gender	Age (years)	Body Mass (kg)	Height (cm)	Diagnosis	AFO	GMFCS
1	M	9	27.5	137.5	Hemi R	sAFO	II
2	F	10	39	143	Hemi L	NHT3	I
3	M	10	23	130	Hemi R	Pull up	II
4	M	6	23	119	Hemi R	sAFO	II
5	M	10	32.5	140	Hemi R	NHT4	I
6	M	10	33	129	Hemi R	sAFO	I
7	F	7	23	116.5	Hemi R	sAFO	II
8	M	7	27	135.5	Hemi R	sAFO	II
9	F	8	31	136.5	Hemi L	sAFO	I
10	M	8	24	129	Hemi L	PLS	II
11	F	7	29	129	Hemi R	sAFO	I
12	M	10	21.5	130	Hemi L	NTH4	II
13	M	8	23	124.5	Hemi L	sAFO	I
14	F	8	37.5	132	Hemi R	sAFO	II
15	M	7	25	126	Hemi R	sAFO	I
16	M	7	28	125	Hemi L	Pull up	II
17	F	5	24	125	Hemi L	sAFO	I
18	F	7	22	120	Hemi L	sAFO	II
MEAN (SD)	[M: F] 11: 7	8.0 (1.5)	27.4 (5.3)	129.3 (7.3)	[R: L] 10: 8		[I: II] 8: 10

**Table 2:** Spatiotemporal parameters for the less affected and affected limb with commonly used AFOs and new generation AFO in the three conditions: downhill (−5°), level-ground walking (0°), and uphill (+10°). Data are reported as the median (interquartile range). In bold, statistically significant differences are shown as determined by the Wilcoxon test in downhill, level-ground, and uphill conditions. GC: gait cycle; iqr: interquartile range.

	Spatio-Temporal Parameters	Side	Median (iqr)		*p*-Value Wilcoxon Test
			Commonly Used AFOs	New Generation AFO	
Downhill (−5°)	Stance phase (%GC)	Less affected	68.84 (2.31)	67.84 (3.02)	0.5862
		Affected	66.64 (3.92)	65.72 (3.86)	0.1930
	Stride time (s)	Less affected	1.21 (0.14)	1.22 (0.15)	0.6192
		Affected	1.20 (0.14)	1.20 (0.14)	0.3812
	Step length (m)	Less affected	0.39 (0.06)	0.36 (0.08)	0.1024
		Affected	0.32 (0.09)	0.33 (0.09)	**0.0497**
	Step width (m)	Less affected	0.18 (0.09)	0.18 (0.07)	0.2097
		Affected	0.18 0.09)	0.18 (0.07)	0.6192
	Stride length (m)	Less affected	0.72 (0.10)	0.73 (0.13)	0.8313
		Affected	0.72 (0.12)	0.74 (0.14)	0.7946
Level-ground (0°)	Stance phase (%GC)	Less affected	69.29 (3.09)	69.52 (1.71)	0.7946
		Affected	66.62 (2.50)	66.52 (2.65)	0.3560
	Stride time (s)	Less affected	1.20 (0.17)	1.21 (0.10)	0.5862
		Affected	1.20 (0.17)	1.22 (0.13)	0.5228
	Step length (m)	Less affected	0.38 (0.07)	0.38 (0.08)	0.5862
		Affected	0.34 (0.10)	0.36 (0.08)	0.3560
	Step width (m)	Less affected	0.17 (0.11)	0.16 (0.06)	0.3318
		Affected	0.16 (0.11)	0.16 (0.06)	0.3318
	Stride length (m)	Less affected	0.73 (0.16)	0.77 (0.20)	0.5228
		Affected	0.73 (0.16)	0.78 (0.20)	0.5862
Uphill (+10°)	Stance phase (%GC)	Less affected	70.95 (6.06)	70.73 (4.60)	0.0684
		Affected	66.59 (2.87)	67.18 (3.36)	**0.0239**
	Stride time (s)	Less affected	1.19 (0.15)	1.20 (0.19)	0.1773
		Affected	1.19 (0.14)	1.20 (0.20)	**0.0495**
	Step length (m)	Less affected	0.34 (0.07)	0.34 (0.10)	0.2097
		Affected	0.37 0.07)	0.36 (0.07)	0.3088
	Step width (m)	Less affected	0.18 (0.10)	0.19 (0.09)	0.4348
		Affected	0.19 (0.10)	0.19 (0.09)	0.5540
	Stride length (m)	Less affected	0.71 (0.15)	0.71 (0.13)	0.1626
		Affected	0.70 (0.14)	0.72 (0.14)	**0.0395**

**Table 3:** Spatiotemporal parameters for the less affected and affected limb in the three conditions: downhill (−5°), level-ground walking (0°), and uphill (+10°). Data are reported as the median (interquartile range). In bold, statistically significant differences are shown as determined by the Friedman test and post-hoc Wilcoxon test with Bonferroni correction. GC: gait cycle; iqr: interquartile range.

	Spatio-Temporal Parameters	Side	Median (iqr)			*p*-Value Friedman Test	*p*-Value (Bonferroni Correction)		
			*Downhill (−5°)*	*Level-Ground (0°)*	*Uphill (+10°)*		*Downhill vs.* *Level-Ground*	*Downhill vs. Uphill*	*Level-Ground* *vs. Uphill*
Commonly used AFOs	Stance phase (%GC)	Less affected	68.84 (2.31)	69.29 (3.09)	70.95 (6.06)	**0.0028**	**0.0352**	**<0.001**	**0.0113**
		Affected	66.64 (3.92)	66.62 (2.50)	66.59 (2.87)	0.1134	-	-	-
	Stride time (s)	Less affected	1.21 (0.14)	1.20 (0.17)	1.19 (0.15)	0.8382	-	-	-
		Affected	1.20 (0.14)	1.20 (0.17)	1.19 (0.14)	0.3902	-	-	-
	Step length (m)	Less affected	0.39 (0.06)	0.38 (0.07)	0.34 (0.07)	**0.0276**	0.4631	**0.0245**	**0.0352**
		Affected	0.32 (0.09)	0.34 (0.10)	0.37 (0.07)	**0.0071**	**0.0352**	**0.0042**	0.0929
	Step width (m)	Less affected	0.18 (0.09)	0.17 (0.11)	0.18 (0.10)	0.1134	-	-	-
		Affected	0.18 0.09)	0.16 (0.11)	0.19 (0.10)	0.0560	-	-	-
	Stride length (m)	Less affected	0.72 (0.10)	0.73 (0.16)	0.71 (0.15)	0.4937	-	-	-
		Affected	0.72 (0.12)	0.73 (0.16)	0.70 (0.14)	0.4655	-	-	-
New Generation AFO	Stance phase (%GC)	Less affected	67.84 (3.02)	69.52 (1.71)	70.73 (4.60)	**<0.001**	**0.0036**	**0.0010**	**0.0277**
		Affected	65.72 (3.86)	66.52 (2.65)	67.18 (3.36)	**0.0136**	0.0840	**0.0113**	**0.0277**
	Stride time (s)	Less affected	1.22 (0.15)	1.21 (0.10)	1.20 (0.19)	**0.0246**	0.0759	0.3812	0.2659
		Affected	1.20 (0.14)	1.22 (0.13)	1.20 (0.20)	**0.0469**	0.1024	0.3088	0.2461
	Step length (m)	Less affected	0.36 (0.08)	0.38 (0.08)	0.34 (0.10)	0.2043	-	-	-
		Affected	0.33 (0.09)	0.36 (0.08)	0.36 (0.07)	**0.0114**	0.1128	**0.0031**	0.3560
	Step width (m)	Less affected	0.18 (0.07)	0.16 (0.06)	0.19 (0.09)	0.1615	-	-	-
		Affected	0.18 (0.07)	0.16 (0.06)	0.19 (0.09)	0.1009	-	-	-
	Stride length (m)	Less affected	0.73 (0.13)	0.77 (0.20)	0.71 (0.13)	0.0560	-	-	-
		Affected	0.74 (0.14)	0.78 (0.20)	0.72 (0.14)	0.2298	-	-	-

## Data Availability

Data is contained within the article or Supplementary Material. The data presented in this study are available in Zenodo. https://doi.org/10.5281/zenodo.10820897.

## Supplementary Materials

The following supporting information can be downloaded at: https://www.mdpi.com/article/10.3390/bioengineering11030280/s1, Figure S1: trunk kinematic; Figure S2: pelvis kinematic; Figures S3 and S4: kinematic—affected side; Figures S5 and S6: kinematic—less affected side; Figures S7 and S8: kinetic—affected side; Figures S9 and S10: kinetic—less affected side; Figures S11 and S12: trunk and pelvis kinematic; Figures S13–S18: lower limb kinematics; Figures S19–24: lower limb kinetics.
