# Supplementary material for: The Effect of a New Generation of Ankle Foot Orthoses on Sloped Walking in Children with Hemiplegia Using the Gait Real Time Analysis Interactive Lab (GRAIL)
Source: Bioengineering (Basel). 2024 Mar 16;11(3):280. doi: 10.3390/bioengineering11030280 (PMC10968429; doi:10.3390/bioengineering11030280)
Supplement: Supplementary file 1 [file bioengineering-11-00280-s001.zip › bioengineering-2878425-supplementary.pdf]

Article

The Effect of a New Generation of Ankle Foot Orthoses on Sloped Walking in Children with Hemiplegia Using the Gait Real Time Analysis Interactive Lab (GRAIL)

Federica Camuncoli [1], Giorgia Malerba [2], Emilia Biffi [2], Eleonora Diella [2], Eugenio Di Stanislao [3], Guerrino Rosellini [3], Daniele Panzeri [2], Luigi Piccinini [2] and Manuela Galli [1,*]

1 Department of Electronics Information Technology and Bioengineering, Politecnico di Milano, 20133 Milan, Italy; federica.camuncoli@mail.polimi.it

2 Scientific Institute IRCCS Eugenio Medea, 23842 Bosisio Parini,, Italy; giorgia.malerba@lanostrafamiglia.it (G.M.); emilia.biffi@lanostrafamiglia.it (E.B.); eleonora.diella@lanostrafamiglia.it (E.D.); daniele.panzeri@lanostrafamiglia.it (D.P.); luigi.piccinini@lanostrafamiglia.it (L.P.)

3 ITOP SpA Officine Ortopediche, 00036 Palestrina, Italy; distanislao.e@itop.it (E.D.S.); ing.rosellini@itop.it (G.R.)

* Correspondence: manuela.galli@polimi.it

SUPPLEMENTARY MATERIALS

Trunk KINEMATIC

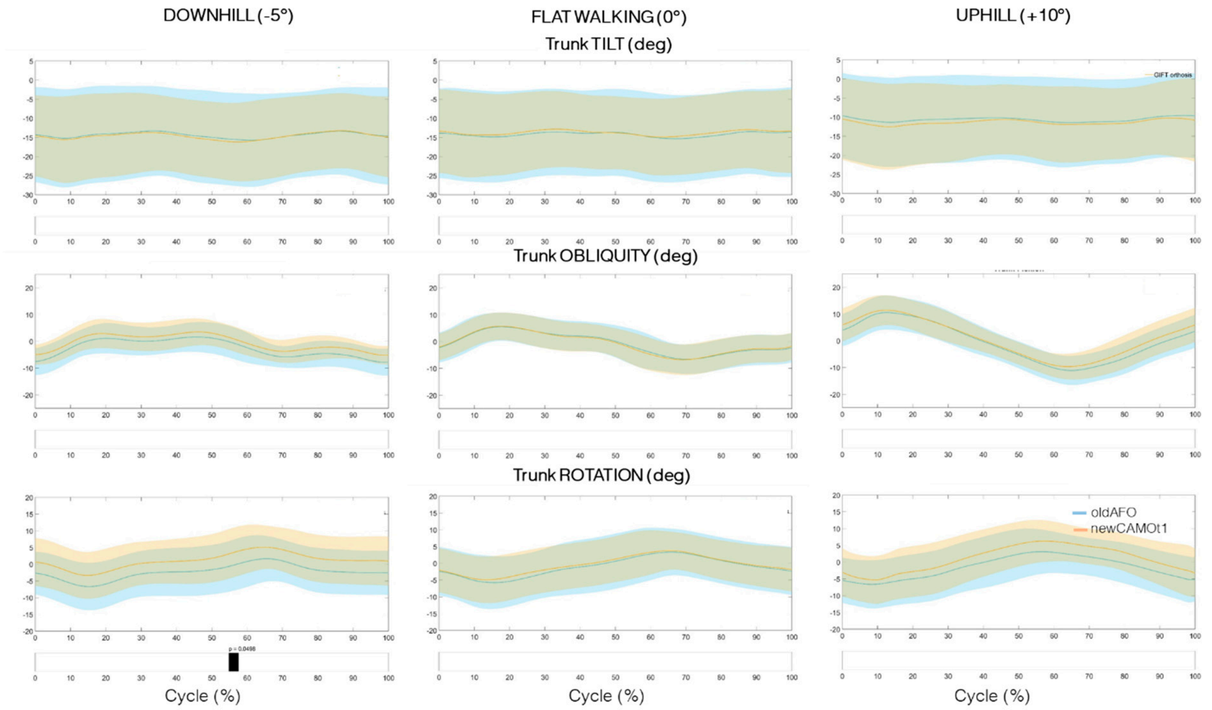

Figure S1. Mean and standard deviation of trunk kinematics in the sagittal, frontal, and transverse planes for commonly used AFOs (depicted in blue) and the new AFO (depicted in orange) after the adaptation period with the related SPM analysis.

Pelvis KINEMATIC

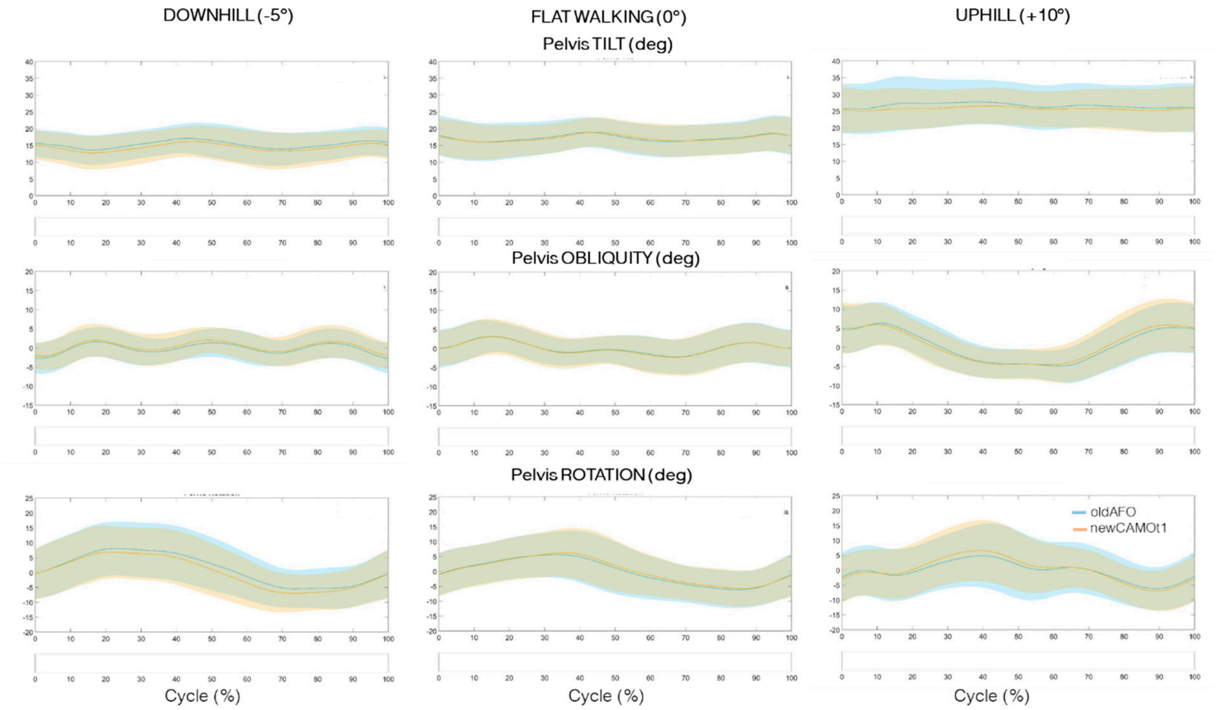

Figure S2. Mean and standard deviation of pelvis kinematics in the sagittal, frontal, and transverse planes for commonly used AFOs (depicted in blue) and the new AFO (depicted in orange) after the adaptation period with the related SPM analysis.

KINEMATIC – AFFECTED SIDE

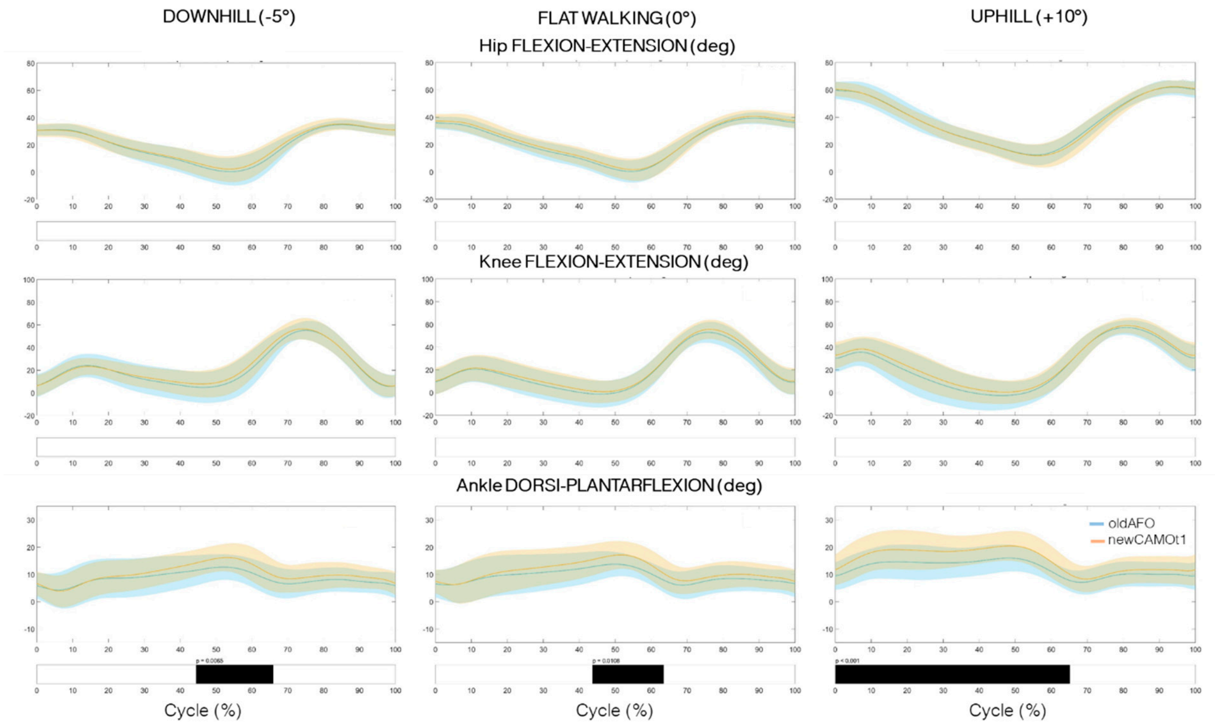

Figure S3. Mean and standard deviation of the kinematics of the affected hip, knee, and ankle joints in the sagittal plane, hip for commonly used AFOs (depicted in blue) and the new AFO (depicted in orange) after the adaptation period with the related SPM analysis.

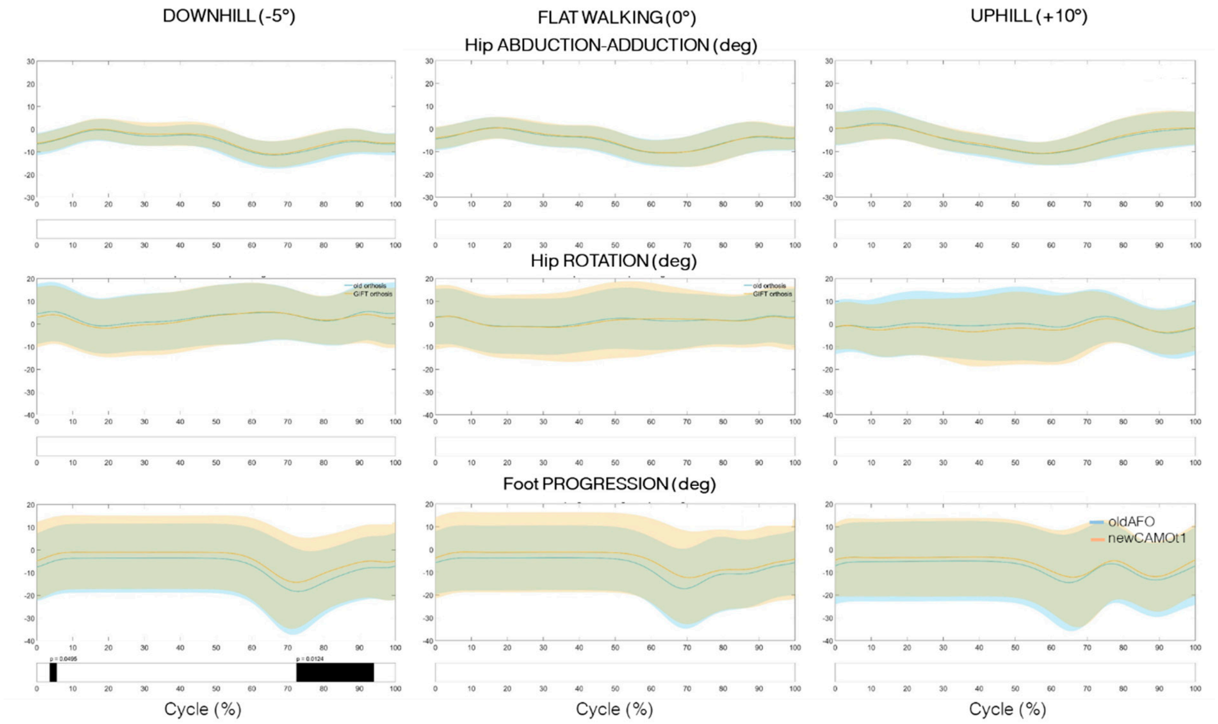

Figure S4. Mean and standard deviation of the kinematics of the affected hip joint abduction and adduction, rotation, and foot progression for commonly used AFOs (depicted in blue) and the new AFO (depicted in orange) after the adaptation period with the related SPM analysis.

KINEMATIC – LESS AFFECTED SIDE

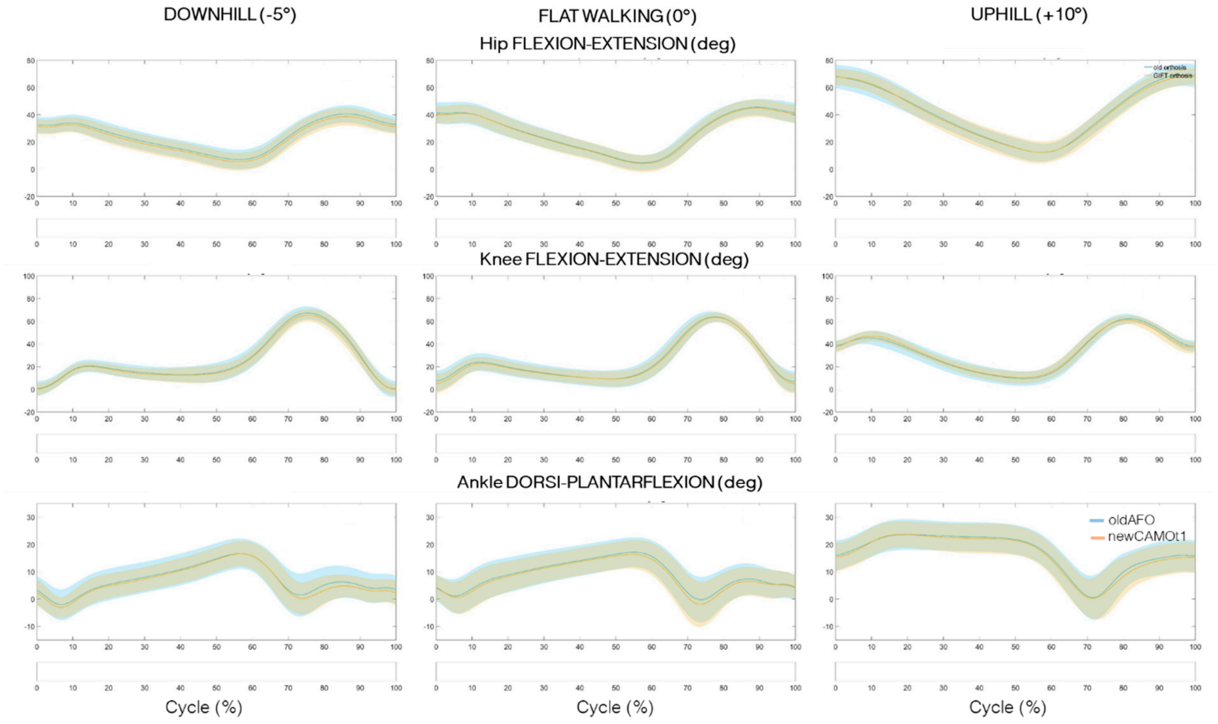

Figure S5. Mean and standard deviation of the kinematics of the less affected hip, knee, and ankle joints in the sagittal plane for commonly used AFOs (depicted in blue) and the new AFO (depicted in orange) after the adaptation period with the related SPM analysis.

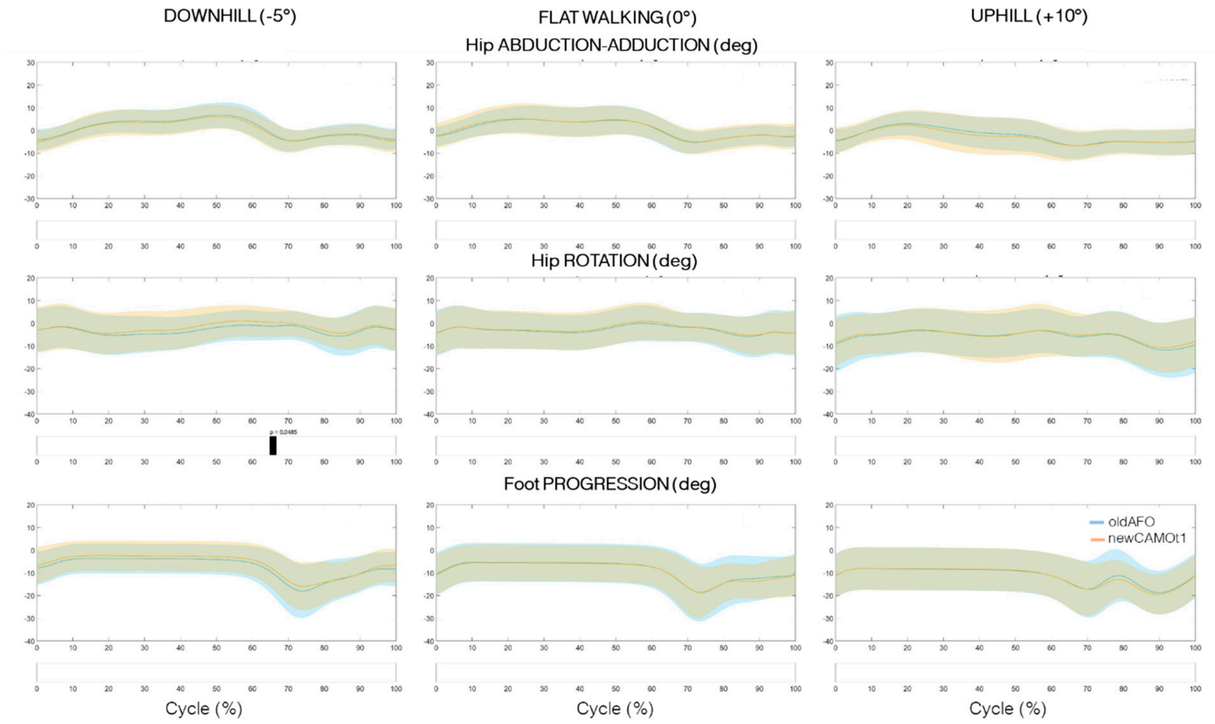

Figure S6. Mean and standard deviation of the kinematics of the less affected hip abduction and adduction, hip rotation, and foot progression for commonly used AFOs (depicted in blue) and the new AFO (depicted in orange) after the adaptation period with the related SPM analysis.

KINETIC – AFFECTED SIDE

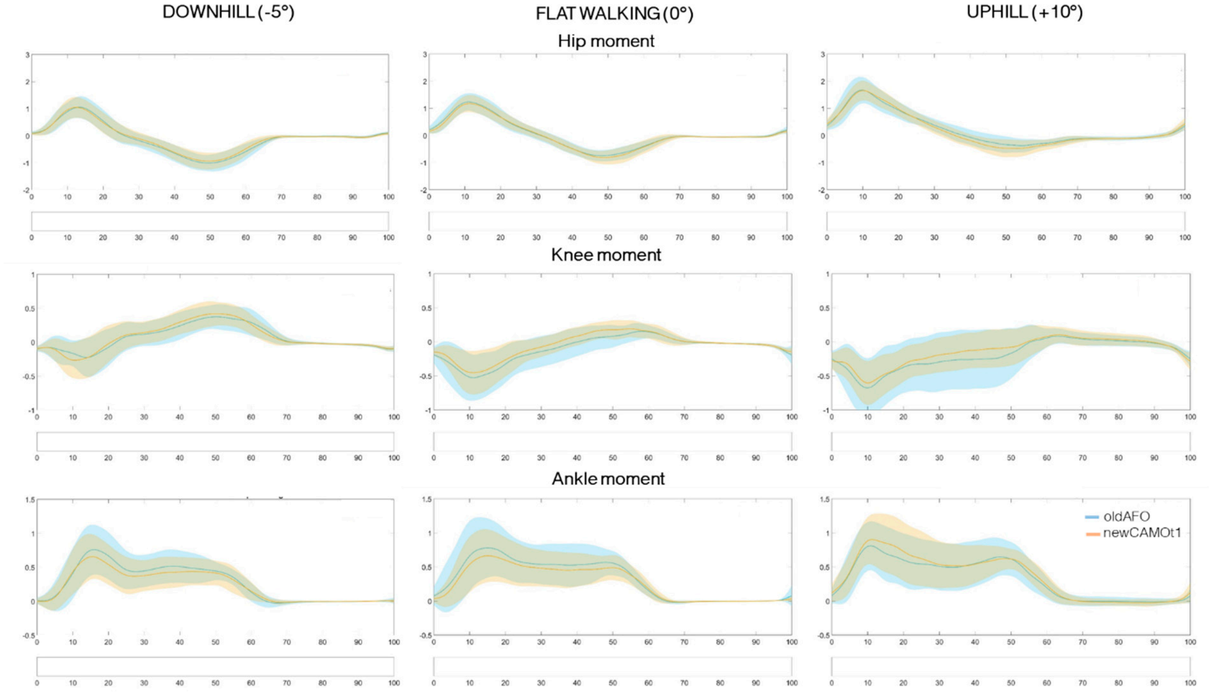

Figure S7. Mean and standard deviation of the moment of the affected hip, knee, and ankle joints in the sagittal plane for commonly used AFOs (depicted in blue) and the new AFO (depicted in orange) after the adaptation period with the related SPM analysis.

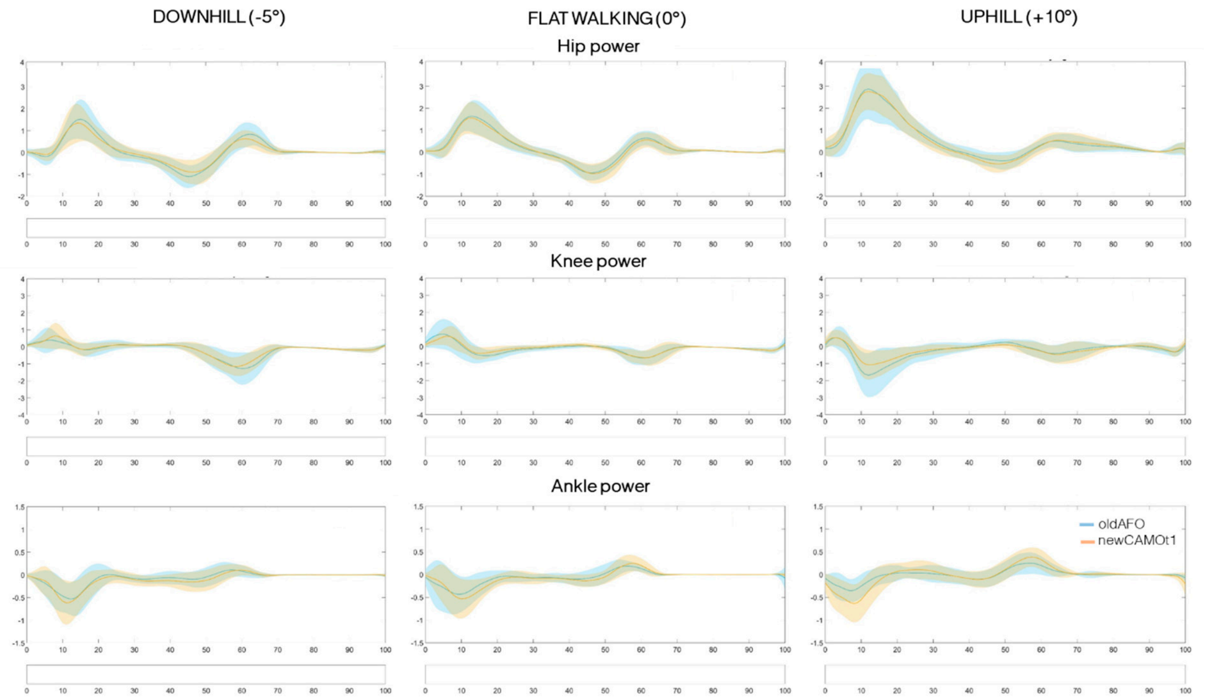

Figure S8. Mean and standard deviation of the power of the affected hip, knee, and ankle joints in the sagittal plane for commonly used AFOs (depicted in blue) and the new AFO (depicted in orange) after the adaptation period with the related SPM analysis.

KINETIC – LESS AFFECTED

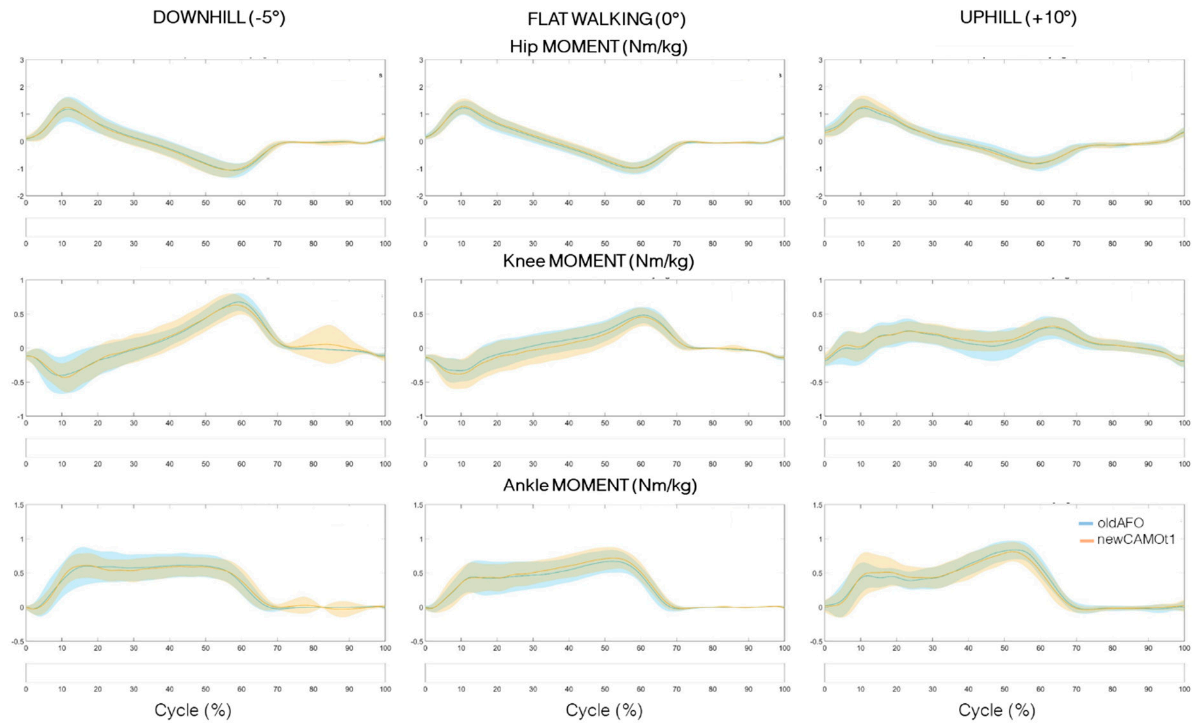

Figure S9. Mean and standard deviation of the moment of the less affected hip, knee, and ankle joints in the sagittal plane for commonly used AFOs (depicted in blue) and the new AFO (depicted in orange) after the adaptation period with the related SPM analysis.

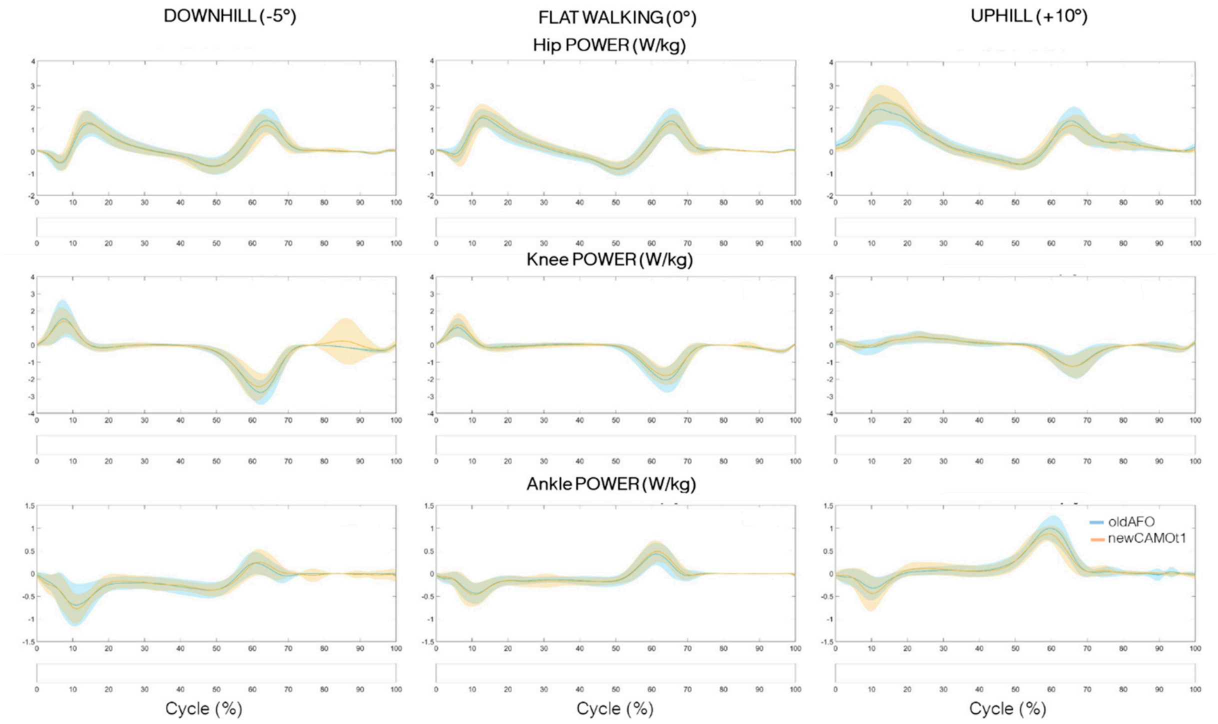

Figure S10. Mean and standard deviation of the power of the less affected hip, knee, and ankle joints in the sagittal plane for commonly used AFOs (depicted in blue) and the new AFO (depicted in orange) after the adaptation period with the related SPM analysis.

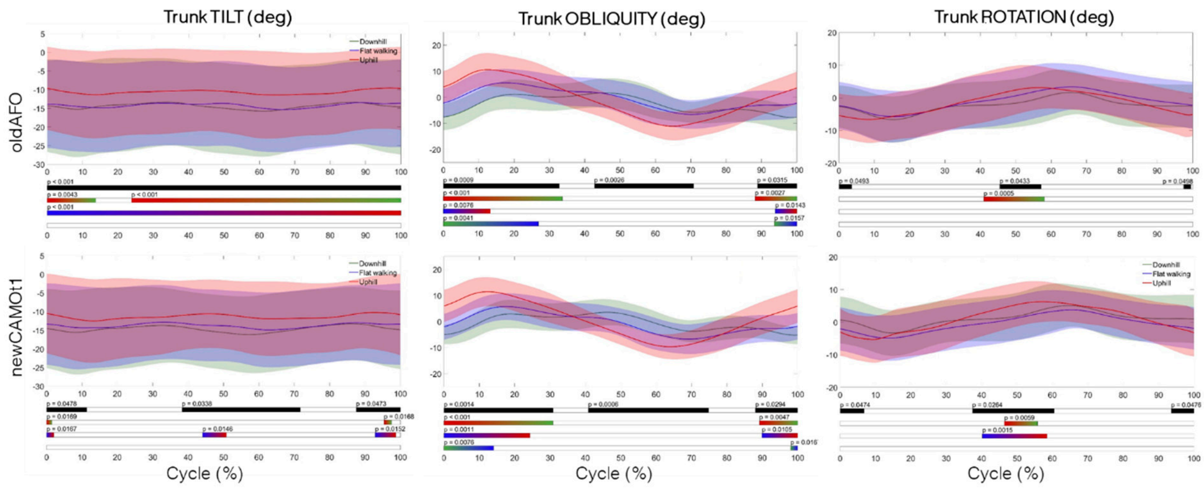

Figure S11. Mean and standard deviation of trunk kinematics in the sagittal, frontal, and transverse planes for commonly used AFOs (oldAFO) and the new AFO (newCAMOt1) downhill (in green), level-groundwalking (in blue) and uphill (in red) with the related SPM analysis.

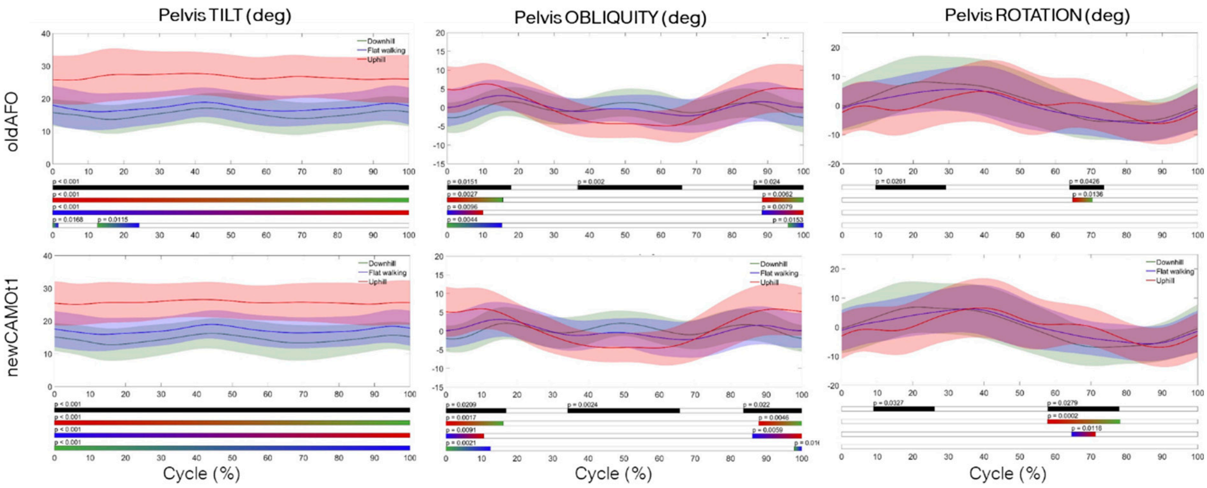

Figure S12. Mean and standard deviation of pelvis kinematics in the sagittal, frontal, and transverse planes for commonly used AFOs (oldAFO) and the new AFO (newCAMOt1) downhill (in green), level-groundwalking (in blue) and uphill (in red) with the related SPM analysis.

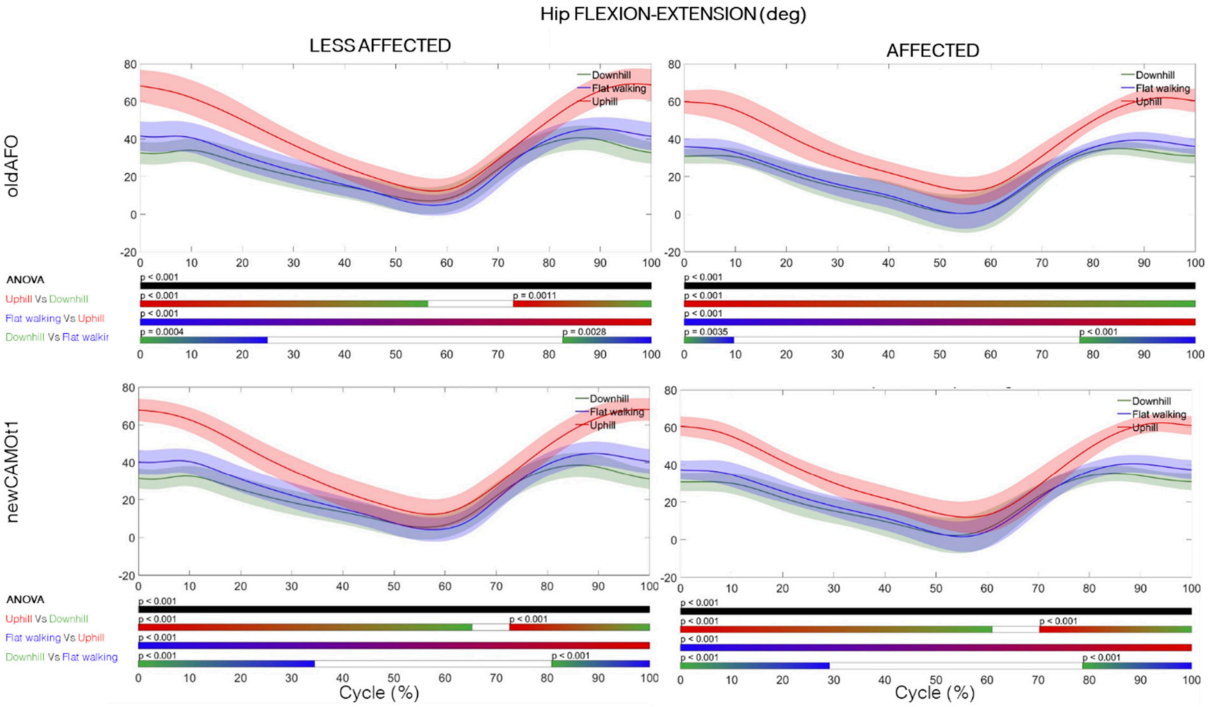

Figure S13. Mean and standard deviation of hip kinematics in the sagittal plane for the affected and less affected sides with commonly used AFOs (oldAFO) and the new AFO (newCAMOt1) during downhill (in green), level-groundwalking (in blue), and uphill (in red) conditions, along with the corresponding SPM analysis.

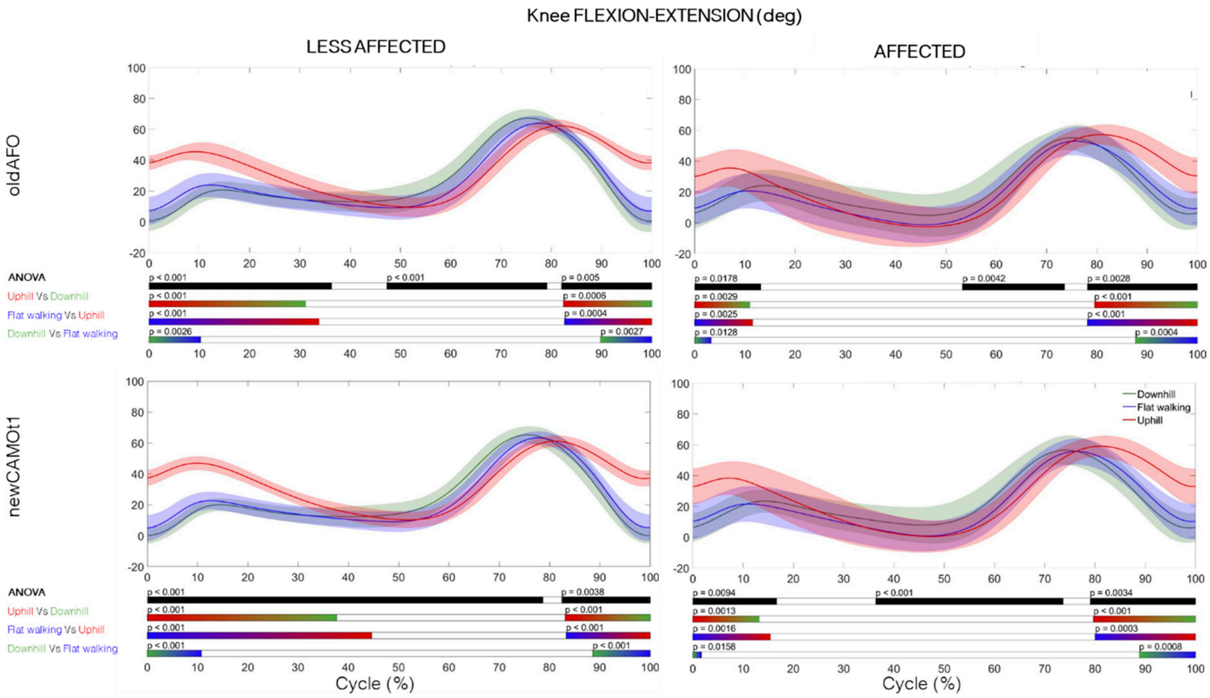

Figure S14. Mean and standard deviation of knee kinematics in the sagittal plane for the affected and less affected sides with commonly used AFOs (oldAFO) and the new AFO (newCAMOt1) during downhill (in green), level-groundwalking (in blue), and uphill (in red) conditions, along with the corresponding SPM analysis.

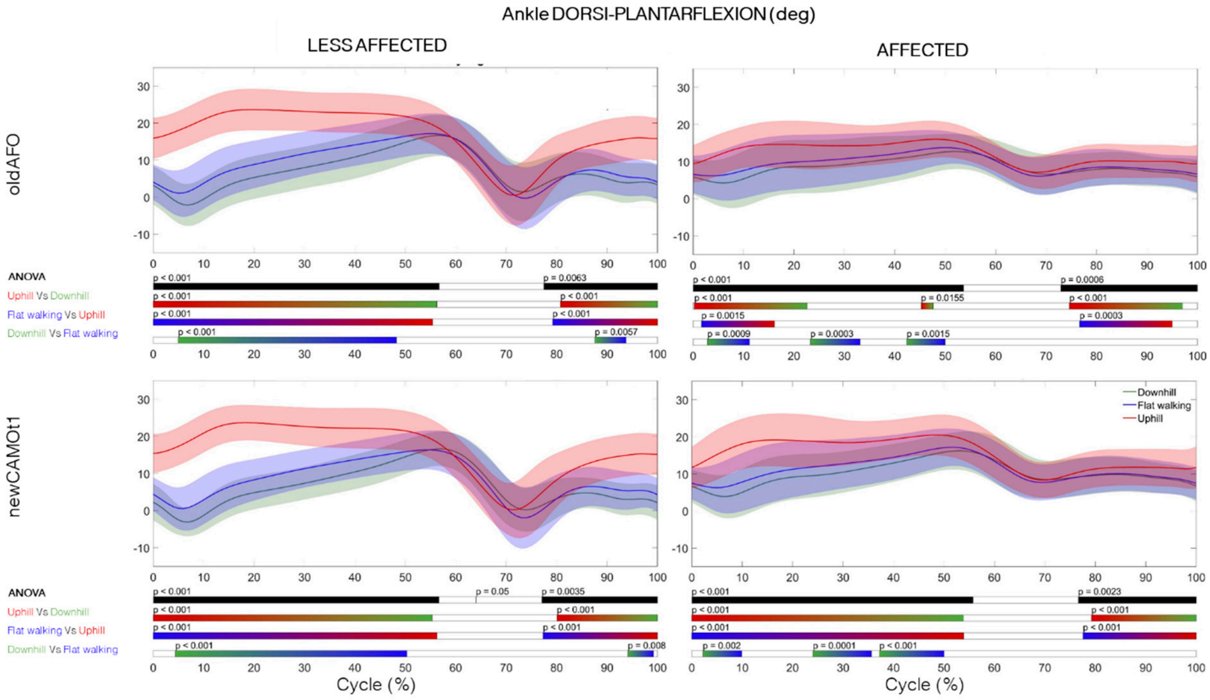

Figure S15. Mean and standard deviation of ankle kinematics in the sagittal plane for the affected and less affected sides with commonly used AFOs (oldAFO) and the new AFO (newCAMOt1) during downhill (in green), level-groundwalking (in blue), and uphill (in red) conditions, along with the corresponding SPM analysis.

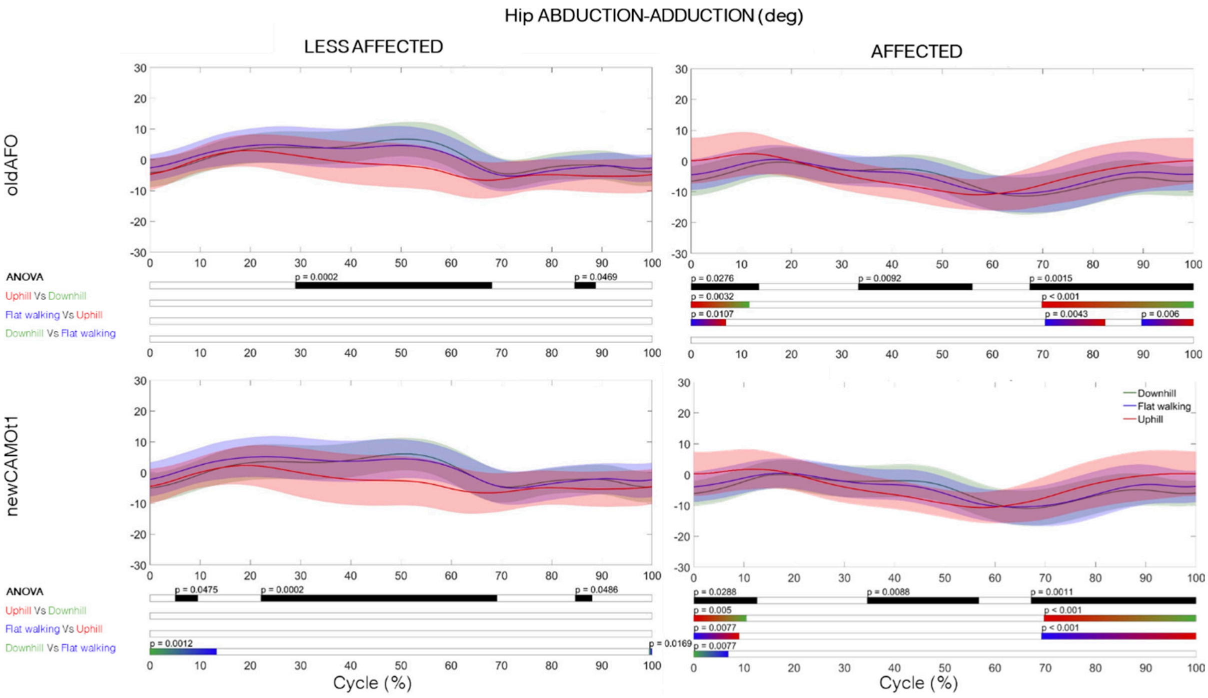

Figure S16. Mean and standard deviation of hip kinematics in the frontal plane for the affected and less affected sides with commonly used AFOs (oldAFO) and the new AFO (newCAMOt1) during downhill (in green), level-groundwalking (in blue), and uphill (in red) conditions, along with the corresponding SPM analysis.

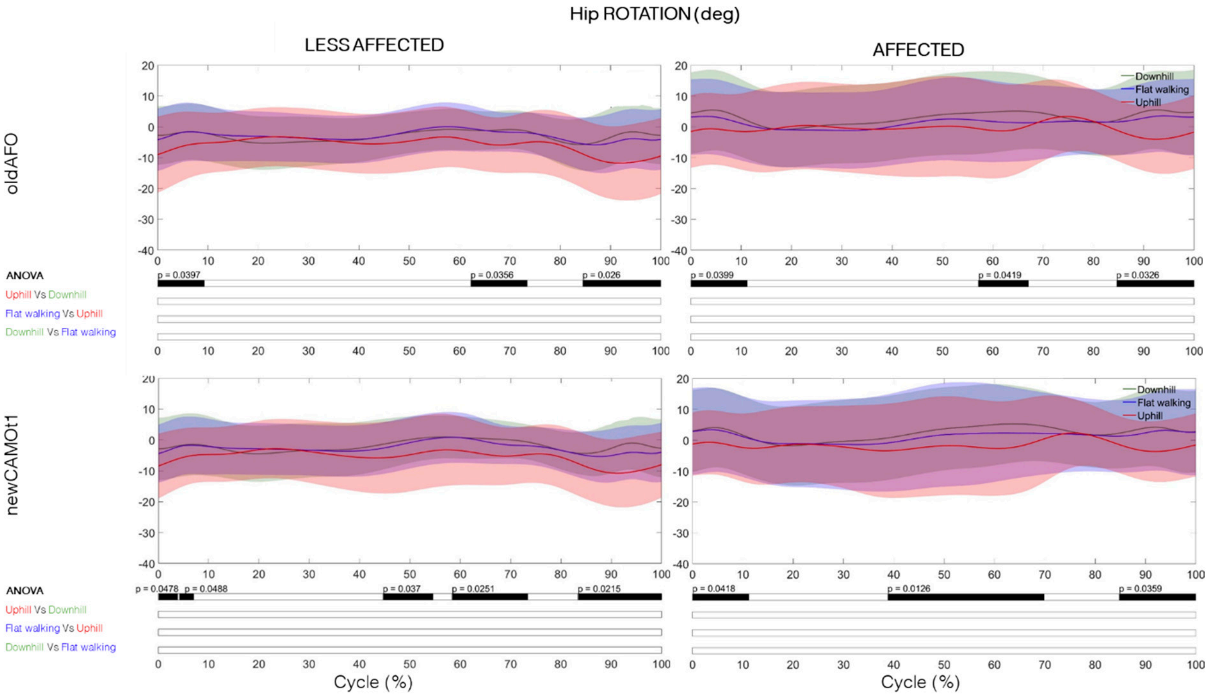

Figure S17. Mean and standard deviation of hip kinematics in the transversal plane for the affected and less affected sides with commonly used AFOs (oldAFO) and the new AFO (newCAMOt1) during downhill (in green), level-groundwalking (in blue), and uphill (in red) conditions, along with the corresponding SPM analysis.

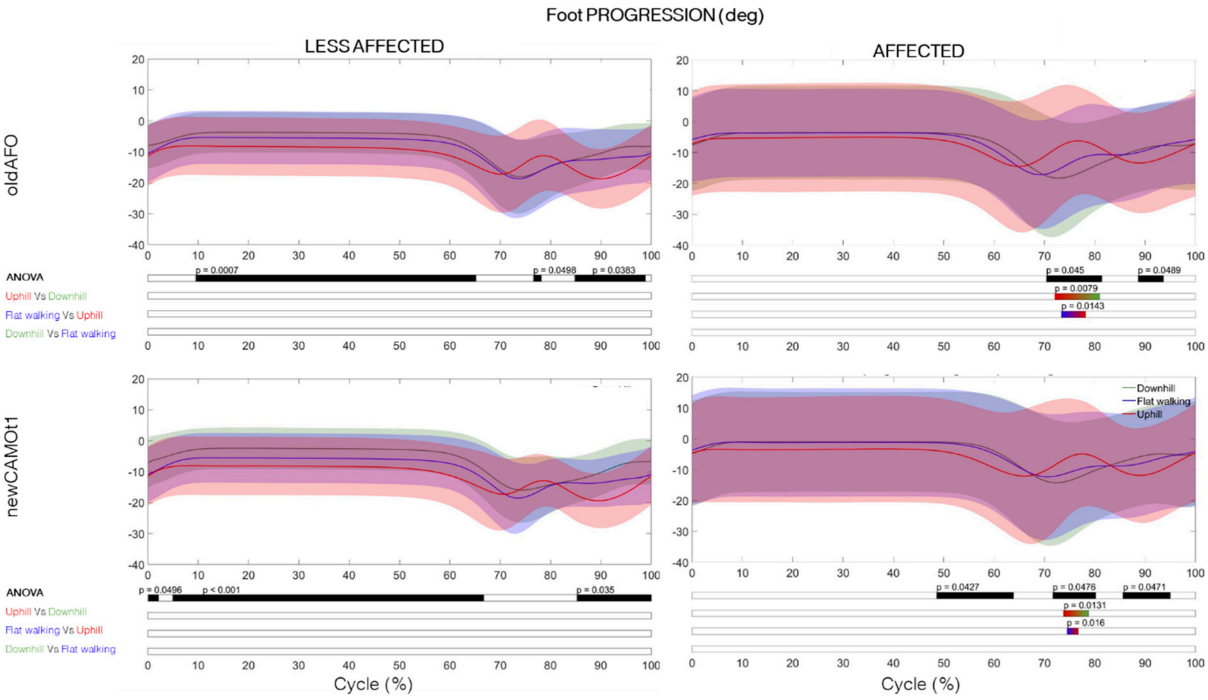

Figure S18. Mean and standard deviation of the foot progression for the affected and less affected sides with commonly used AFOs (oldAFO) and the new AFO (newCAMOt1) during downhill (in green), level-groundwalking (in blue), and uphill (in red) conditions, along with the corresponding SPM analysis.

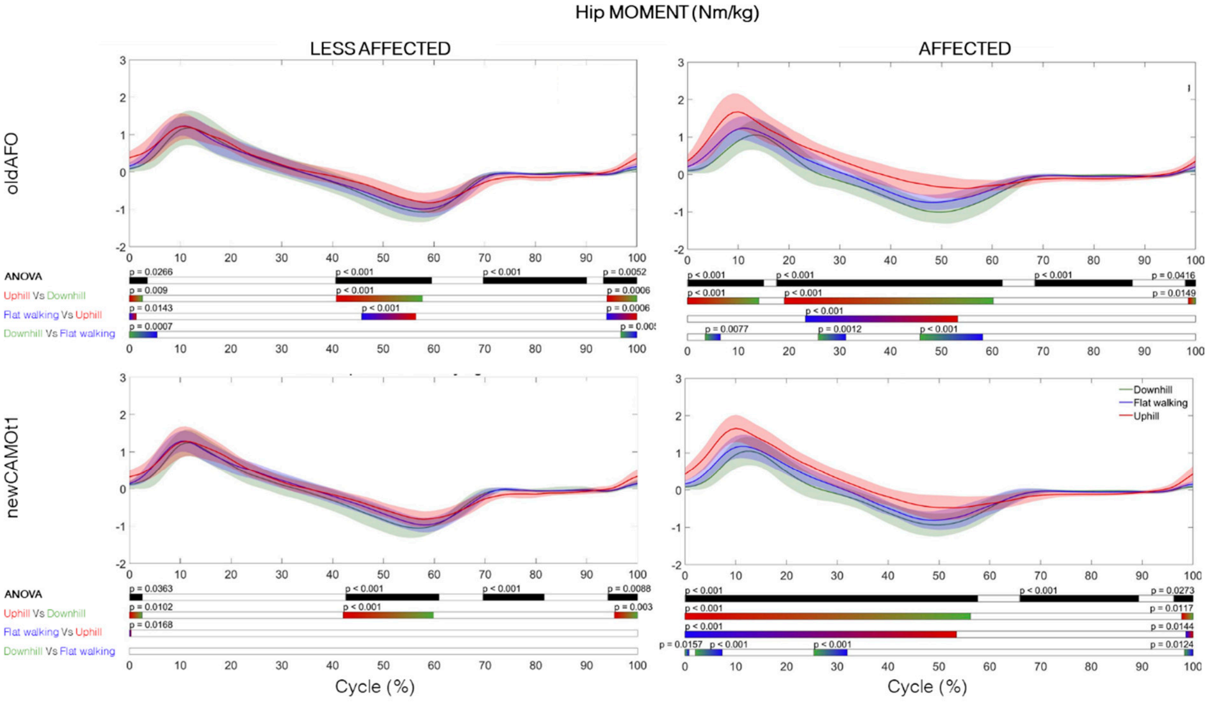

Figure S19. Mean and standard deviation of hip moment in the sagittal plane for the affected and less affected sides with commonly used AFOs (oldAFO) and the new AFO (newCAMOt1) during

downhill (in green), level-groundwalking (in blue), and uphill (in red) conditions, along with the corresponding SPM analysis.

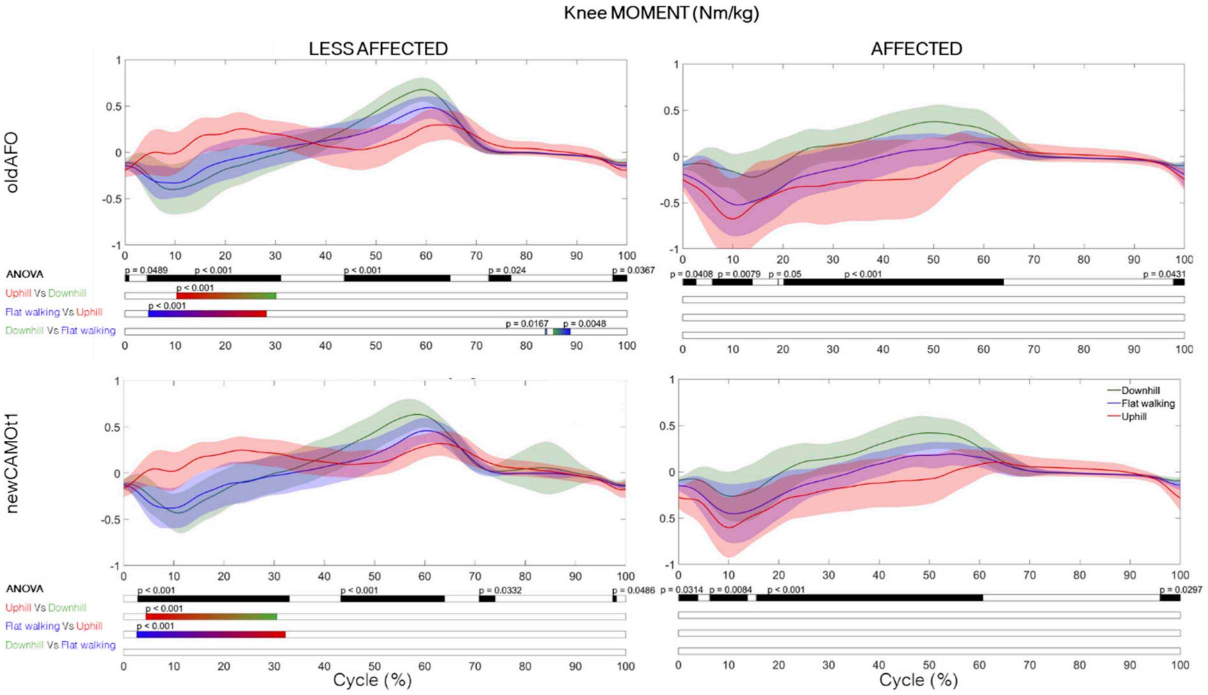

Figure S20. Mean and standard deviation of knee moment in the sagittal plane for the affected and less affected sides with commonly used AFOs (oldAFO) and the new AFO (newCAMOt1) during downhill (in green), level-groundwalking (in blue), and uphill (in red) conditions, along with the corresponding SPM analysis.

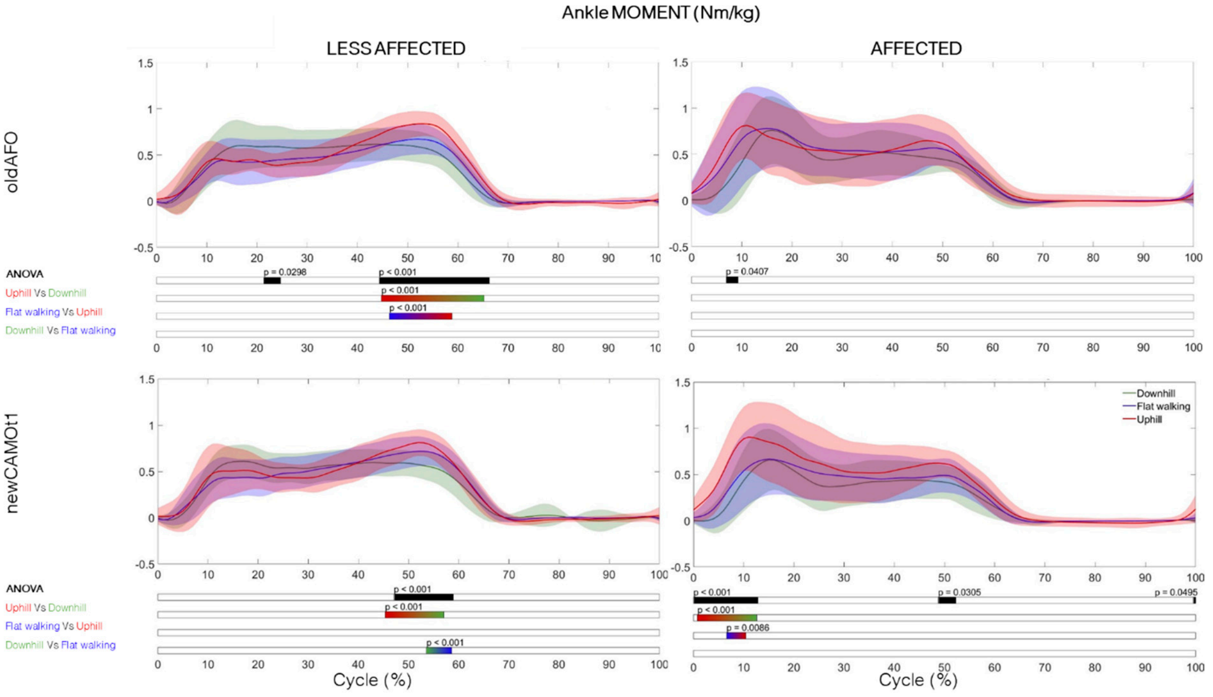

Figure S21. Mean and standard deviation of ankle moment in the sagittal plane for the affected and less affected sides with commonly used AFOs (oldAFO) and the new AFO (newCAMOt1) during downhill (in green), level-groundwalking (in blue), and uphill (in red) conditions, along with the corresponding SPM analysis.

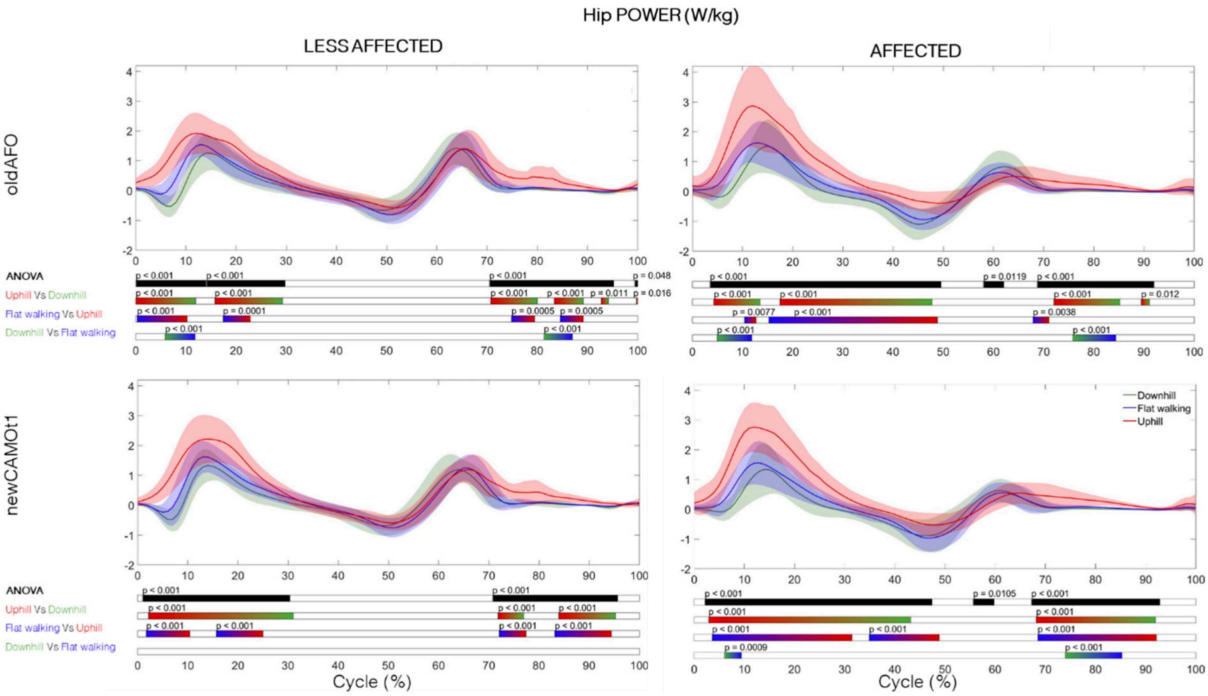

Figure S22. Mean and standard deviation of hip power in the sagittal plane for the affected and less affected sides with commonly used AFOs (oldAFO) and the new AFO (newCAMOt1) during downhill (in green), level-ground walking (in blue), and uphill (in red) conditions, along with the corresponding SPM analysis.

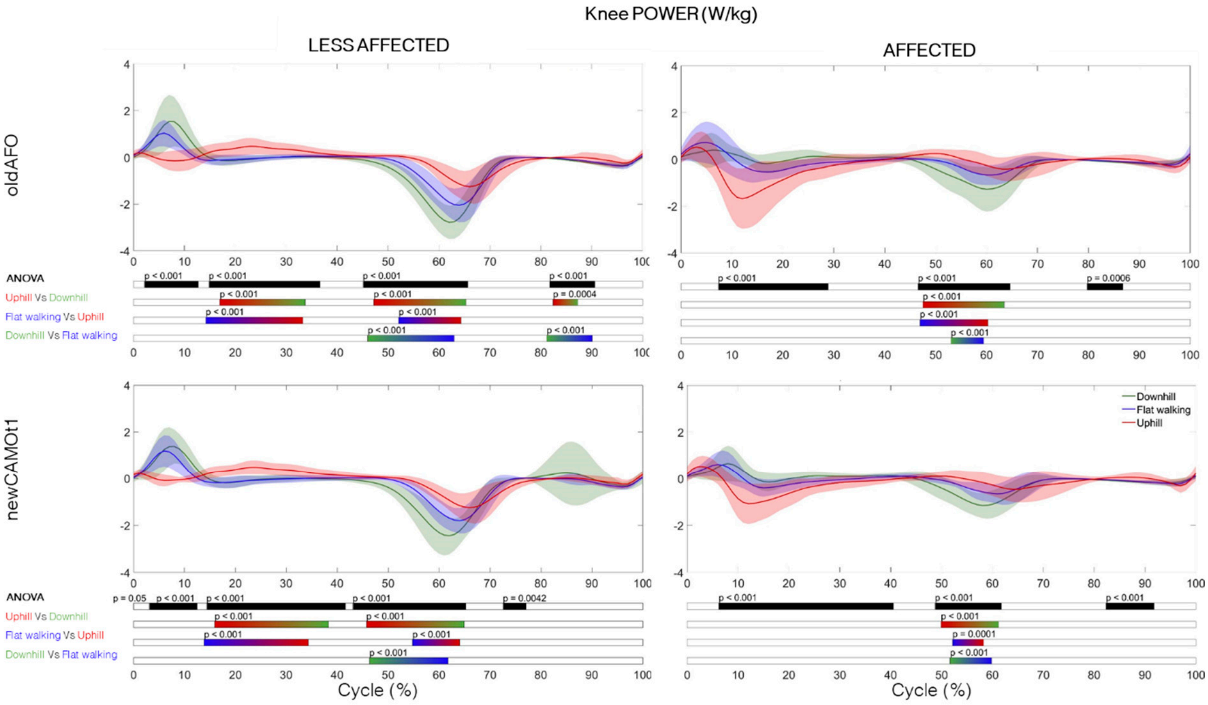

Figure S23. Mean and standard deviation of knee power in the sagittal plane for the affected and less affected sides with commonly used AFOs (oldAFO) and the new AFO (newCAMOt1) during downhill (in green), level-ground walking (in blue), and uphill (in red) conditions, along with the corresponding SPM analysis.

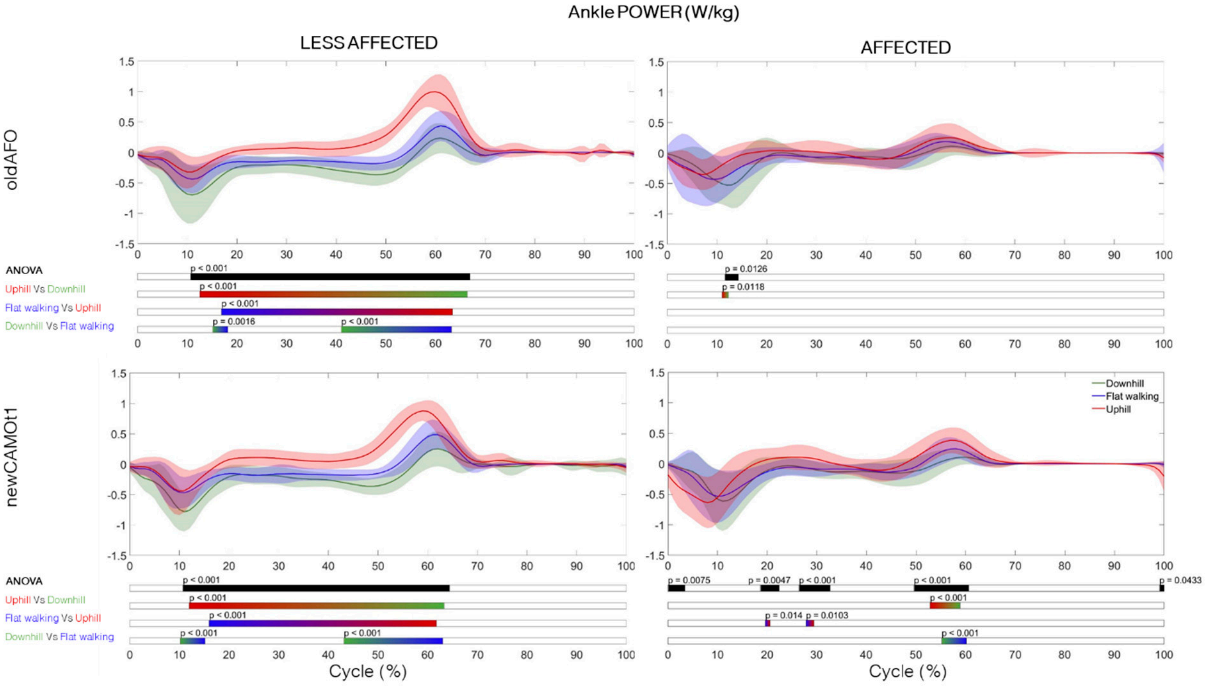

Figure S24. Mean and standard deviation of ankle power in the sagittal plane for the affected and less affected sides with commonly used AFOs (oldAFO) and the new AFO (newCAMOt1) during downhill (in green), level-groundwalking (in blue), and uphill (in red) conditions, along with the corresponding SPM analysis.
